# Structures, fundamental properties, and potential applications of low-dimensional C_60_ polymers and other nanocarbons: a review

**DOI:** 10.1080/14686996.2024.2346068

**Published:** 2024-05-20

**Authors:** Jun Onoe, Yusuke Noda, Qian Wang, Koji Harano, Masato Nakaya, Tomonobu Nakayama

**Affiliations:** a Department of Energy Science and Engineering, Nagoya University, Nagoya, Japan; b Department of Information and Communication Engineering, Okayama Prefectural University, Soja, Japan; c School of Materials Science and Engineering/Center for Applied Physics and Technology, Peking University, Beijing, China; d Center for Basic Research on Materials, and Division of International Collaborations and Public Relations, National Institute for Materials Science (NIMS), Tsukuba, Japan

**Keywords:** Low dimensional nanocarbons, structures, fundamental properties, potential applications

## Abstract

Since carbon (C) atom has a variety of chemical bonds *via* hybridization between s and p atomic orbitals, it is well known that there are robust carbon materials. In particular, discovery of C_60_ has been an epoch making to cultivate nanocarbon fields. Since then, nanocarbon materials such as nanotube and graphene have been reported. It is interesting to note that C_60_ is soluble and volatile unlike nanotube and graphene. This indicates that C_60_ film is easy to be produced on any kinds of substrates, which is advantage for device fabrication. In particular, electron-/photo-induced C_60_ polymerization finally results in formation of one-dimensional (1D) metallic peanut-shaped and 2D dumbbell-shaped semiconducting C_60_ polymers, respectively. This enables us to control the physicochemical properties of C_60_ films using electron-/photo-lithography techniques. In this review, we focused on the structures, fundamental properties, and potential applications of the low-dimensional C_60_ polymers and other nanocarbons such as C_60_ peapods, wavy-structured graphene, and penta-nanotubes with topological defects. We hope this review will provide new insights for producing new novel nanocarbon materials and inspire broad readers to cultivate new further research in carbon materials.

## IMPACT STATEMENT

We review the structures, fundamental properties, and applications of low-dimensional C_60_ polymers and other related nanocarbons such as C_60_ peapods, wavy-structured graphene, and penta-nanotubes from a standpoint of topological defects.

## Graphical abstract

**Figure:**
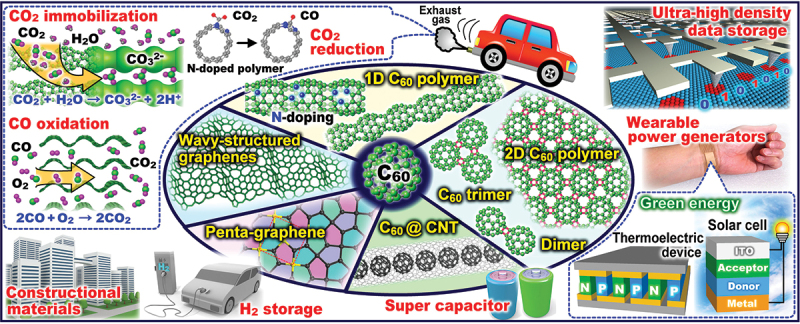

## 1. Introduction

Since the discovery of low-dimensional carbon materials such as zero-dimensional C_60_ [1], one-dimensional (1D) carbon nanotubes (CNTs) [2], and 2D graphene [3] from the end of the 20th century to the beginning of the 21st century, they are well known to have cultivated new science and industrial applications so far [4–6].

**Figure 1.**
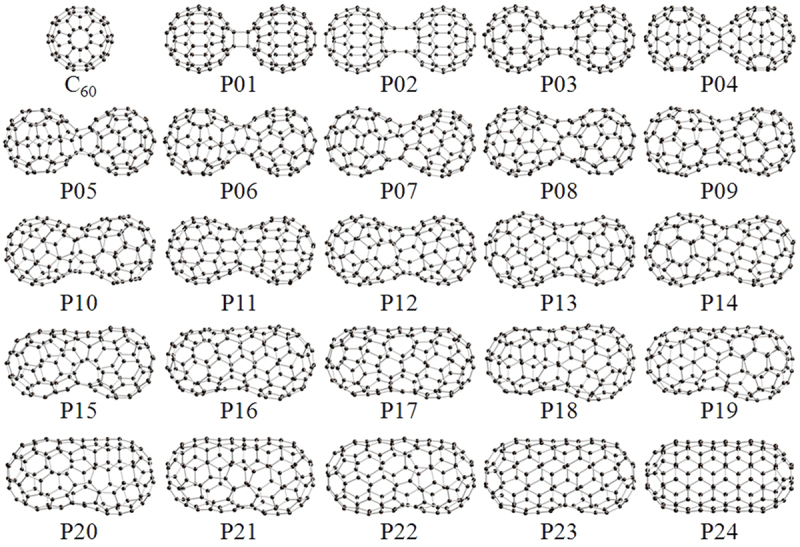
Schematic representation of the change in geometrical structure from C_60_ dimer (P01) to C_120_ nanotube (P24) *via* the general Stone-Wales transformation [8]. The structures of P01–P24 derived from the GSW transformation [7] were geometrically optimized using Gaussian03 package [9].

Since C_60_ has unique features such as solubility (against organic solvents such as toluene) and volatility (Chap. 5 of Ref. [4]), it is easy to produce C_60_ films on any substrate using spin-coat or thermal evaporation method, which is advantage for device fabrication. In addition, the electric conductivity of C_60_ films can be controlled from insulating to semiconducting/metallic using photo/electron-induced polymerization (Chap. 7 of Ref. [4]) with a variety of cross-linked structures based on the generalized Stone-Wales (GSW) transformation [7] in association with topological defects. Figure 1 shows schematic representation of the change in geometrical structure from dumbbell-shaped C_60_ dimer to C_120_ nanotube *via* the GSW transformation [7] in association with topological defects. Although the other polymerization methods such as high-pressure/high-temperature (HPHT) treatment [10], alkali-metal doping [11], and mechanochemical reaction [12] are also well known so far, these methods are however hard to be employed practically in device fabrication processes. On the other hand, since photolithography and electron-beam (EB) lithography are well known to be a standard technique for many semiconductor devices, the modification of the physicochemical properties by photo/electron-induced polymerization is useful to improve the device performance such as solar cells [13].

Takashima et al. [8]; licensed under a Creative Commons Attribution (CC BY) license.

**Figure 2.**
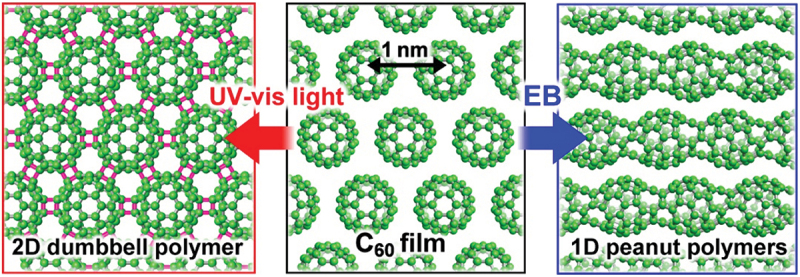
Schematic illustration of pristine C_60_ film (center), 2D semiconducting dumbbell-shaped C_60_ polymer network (left), and 1D metallic uneven-structured (peanut-shaped) C_60_ polymer chain (right) [20].

From this standpoint, Onoe et al. have hitherto investigated the structural and physicochemical properties of photo- and electron-beam (EB)-induced polymerized C_60_ films under ultrahigh vacuum (UHV) conditions [14,15], and demonstrated that photopolymerization finally results in formation of a 2D semiconducting dumbbell-shaped C_60_ polymer [16–20], whereas EB-polymerization finally results in formation of a 1D metallic uneven-structured (peanut-shaped) C_60_ polymer [21–26] *via* the GSW transformation [7], as shown in Figure 2 [20].

Onoe et al. [20]; licensed under a Creative Commons Attribution (CC BY) license.

In this review, we will introduce the structures, fundamental properties, and potential applications of 1D peanut-shaped C_60_ polymer and related materials formed *via* electron-induced polymerization in Section 2, and those of 2D dumbbell-shaped C_60_ polymer and related materials formed *via* photo-induced polymerization in Section 3. Thereafter, we will introduce the other low-dimensional nanocarbon materials such as C_60_ peapods, wavy-structured graphenes, and penta-nanotubes in Section 4. Finally, we will describe the outlook for future research in nanocarbon materials in Section 5. Although nanodiamonds are regarded as quantum dots, there have been many review papers so far [27–29]. Thus, they are omitted in this review.

## 2. One-dimensional peanut-shaped C_60_ polymers and related nanocarbons formed *via* electron-induced polymerization

### 2.1. Structures

Onoe et al. have examined the cross-linked structure of 1D C_60_ polymer using *in situ* Fourier-transform infrared (FT-IR) spectroscopy in combination with first-principles calculations based on density functional theory (DFT) [8]. Comparison between the experimental and theoretical IR spectra suggests that the cross-linked structure of the 1D peanut-shaped C_60_ polymer is roughly close to that of P08 *via* intermediate cross-linkages of P01, P04, and P06 (see Figure 1) [30]. Since the IR peaks originating from the 1D polymer are intense and narrow (the full width at half maximum is almost same as that of pristine C_60_ film), the cross-linkage is considered to be one given structure.

**Figure 3.**
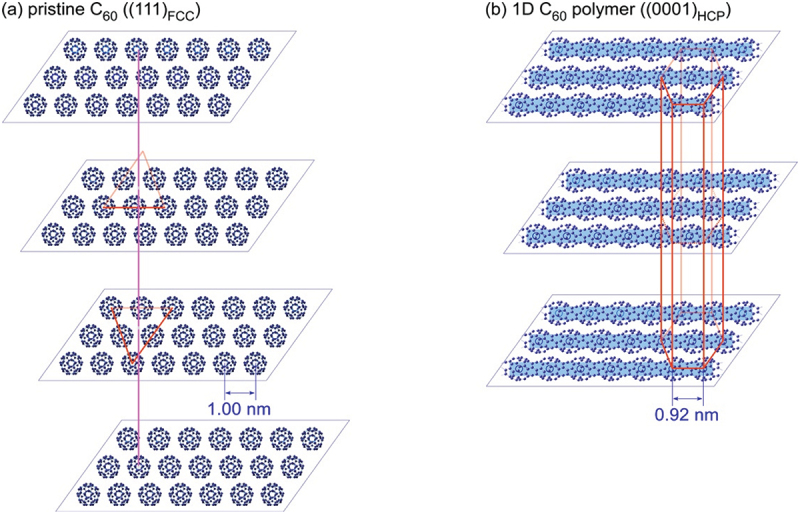
Schematic illustration of (a) face-centered-cubic (FCC) C_60_ structure model and (b) hexagonal-closed-pack (HCP) structure model for 1D C_60_ polymer [26]. Here, blue band lines shown in FIG. (b) Show the direction of 1D C_60_–C_60_ polymerization.

**Figure 4.**
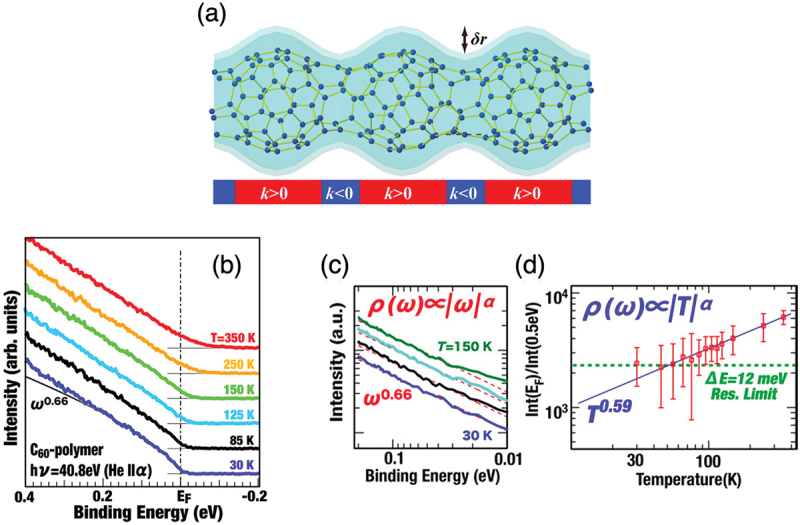
(a) Schematic illustration of the 1D metal C_60_ polymer with an uneven peanuts-shaped structure similar to the cross-linked structure of the P08 C_120_ stable isomer shown in Figure 1. The area colored in sky blue represents a geometrical curved surface in which π-electrons move one-dimensionally. The *δr* denotes the degree of uneven deformation. (b) Temperature dependence of the photoelectron spectra of the 1D peanut-shaped C_60_ polymer in the vicinity of *E*_F_. (c,d) The power-law dependence of the photoelectron spectral function shown in (b) on the binding energy (c) and temperature (d) [24].

Masuda et al. further examined the 1D polymer obtained from electron-beam irradiated C_60_ single crystal (SC) films using high-resolution transmittance electron microscope (HRTEM). Judging from the cryo-HRTEM images and their corresponding fast Fourier-transformed (FFT) patterns [26] in comparison with the results of electron diffraction pattern [25], they demonstrated the structural change from pristine C_60_ SC film to 1D C_60_ polymer film by EB irradiation, as shown in Figure 3. Here, the unit cell of (a) and (b) is shown by a red solid line. C_60_ molecules in the three-fold symmetrical [111]_FCC_ layer (Figure 3(a)) are polymerized to form the 1D C_60_ polymer [0001]_HCP_ with an intermolecular distance of 0.93 nm along one of the three nearest neighboring directions of [−12–10]_HCP_ in association with the symmetry broken to change the stacking from FCC to HCP-m (Figure 4(b)). Here, FCC and HCP are the abbreviation of ‘face-centered-cubic’ and ‘hexagonal-closed-pack’, respectively.

Masuda et al. [26]; licensed under a Creative Commons Attribution (CC BY) license.

Onoe et al. [24]; licensed under a Creative Commons Attribution (CC BY) license.

### 2.2. Fundamental properties

The 1D peanut-shaped C_60_ polymer with a concave-convex periodic curved structure exhibited physical properties arising from 1D metal such as Peierls transition [31], charge-density-wave phonon anomaly [32], and 1D van Hove singularity of phonon dispersion [33]. Especially, it is interesting to note that the 1D polymer film first demonstrates geometrical curvature effects on the electronic states [24,34], which has been a big puzzle in quantum mechanics since the theoretical predictions in 1950s [35–37].

The behavior of the free electrons on the curved surface is characterized by the Hamilton operator of the following equation (quantum mechanics of sub-manifold),

$$\hat{H}=-\frac{\hbar^{2}}{2m^{*}}\left[\frac{1}{\sqrt{g}}\sum_{i,j=1}^{2}\frac{\partial }{\partial q^{i}}\left(\sqrt{g}g^{\mathrm{ij}}\frac{\partial }{\partial q^{j}}\right)+\left(h^{2}-k\right)\right]$$

Here, m* is the effective mass of the electron, (*q*^1^, *q*^2^) is the curved coordinate system, g^ij^ is the inverse matrix component of matrix [g_ij_], g is the determinant of matrix [g_ij_], and g_ij_ is the metric tensor, respectively. *h* and *k* are the mean curvature and Gaussian curvature, respectively, and represent the degree of curvature at each point on the curved surface. Since *h* and *k* are the functions of g_ij_, the Hamilton operator is uniquely determined by g_ij_ describing a shape once the shape of the surface is determined. It is worth noting that the second term appears as a new scalar potential [Jensen-Koppe-da Costa (JKC) potential] besides the first term corresponding to the kinetic energy of the electron. This is the effective electric field potential caused by the geometric curvature of the surface, and plays a role in driving surface-curvature induced changes in physical properties.

The 1D peanut-shaped polymer exhibits Tomonaga-Luttinger liquid (TLL) states [38,39] which is the direct evidence for 1D metal [24]. Accordingly, the density-of-states (DOS: *ρ*) of 1D metal obey not Fermi-Dirac distribution function but a power-law dependence of binding energy or temperature when measured using photoelectron spectroscopy. Namely,

$$\rho \left(E\right)\propto {\left|E-E_{F}\right|}^{\alpha }\;\mathrm{or}\;\rho \left(E\right)\propto {\left|T\right|}^{\alpha }$$

Here, *E*, *E*_F_, and *T* denote binding energy, Fermi energy, and absolute temperature, respectively. When the exponent α is less than unity (α < 1), it can be regarded as 1D metal with TLL states. Actually, 1D metallic single wall (SW) CNTs show a power-law dependence with an exponent (α) of ca. 0.5 [40].

As shown in Equation 1, to demonstrate whether the JKC potential affects the electronic states or not, Shima et al. first theoretically examined the change in the α using deformed cylinder models (Figure 1 of Ref. [34]), whose radius varies periodically, and found that the variation in the surface curvature inherent to the system gives rise to a significant increase in the power-law exponent of the single-particle DOS (Figure 5 of Ref. [34]).

**Figure 5.**
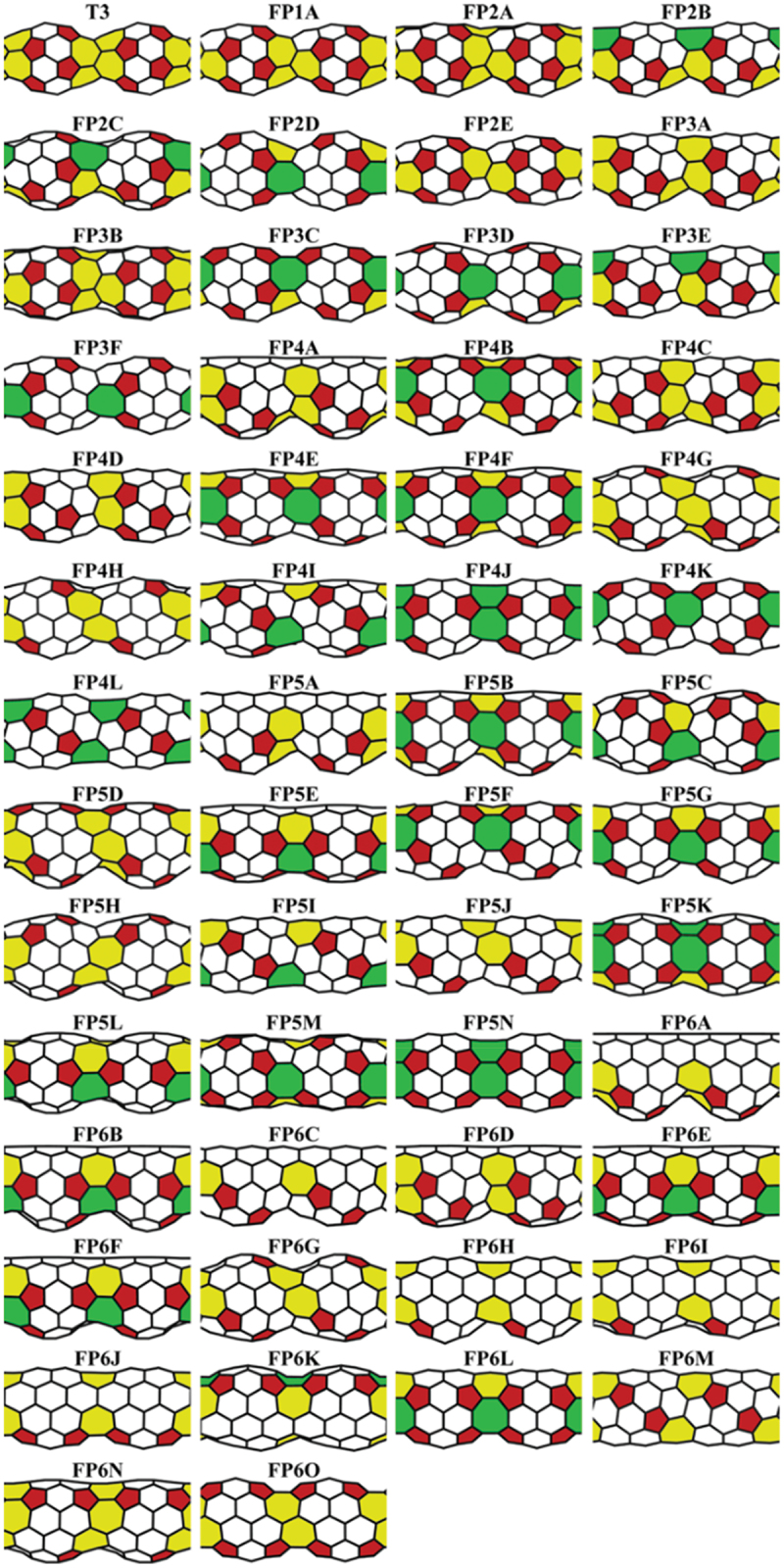
Geometrical structures of 54 different 1D peanuts-shaped C_60_ polymer models. Red, white, yellow, and green carbon-network polygons indicate 5-, 6-, 7-, and 8-membered rings, respectively [41].

Reprinted with permission from Noda et al. [41]. Copyright 2015 American Chemical Society.

Onoe et al. examined the exponent α of 1D peanut-shaped C_60_ polymer film using *in situ* high-resolution photoemission spectroscopy (PES) [24]. As shown in Figure 4(a), the 1D peanut-shaped C_60_ polymer shows a power-law dependence at 30 K, which indicates TLL states in case of 1D metal. By fitting the PES spectra with a power-law function of Equation 2 in the energy range of 18–70 meV, they obtained an exponent *α* of 0.66, as shown in Figure 4(c). Since the TLL exponent α value depends on the choice of an energy range set for fitting, they examined various energy range sets within 18–100 meV (a fitting accuracy of ±0.02) and obtained *α* to be 0.65 ± 0.08. In a similar manner, Figure 4(d) plots the temperature dependence of the ratio of the photoemission intensity at *E*_F_ to the intensity at 0.5 eV in binding energy on a log – log scale, and demonstrates a power-law dependence on temperature in the range of 30–350 K, in which the exponent *α* was obtained to be 0.59 ± 0.04. Judging from the results of Figure 4, the TLL exponent *α* for the 1D peanut-shaped C_60_ polymer can be concluded to be ca. 0.6, which is significantly larger than that of ca. 0.5 (0.43–0.54) for metallic SWCNTs [40,42,43]. In the theoretical work [34], when the radial modulation degree (*δr*) of the uneven structure increased from 0 nm (a straight tube) to 0.16 nm (an uneven peanut-shaped tube), the TLL exponent *α* increased from 0.5 to 0.6. As shown in Figure 4(a), the *δr* can be estimated to be ca. 0.14 nm, which is in good agreement with the predicted value. Details of discussion have been described in Ref. [22]. Thus, Onoe et al. first observed the geometrical curvature effects on electronic states that have been a big puzzle since 1950s. This is a novel property different from that of fullerenes, CNTs, and graphene.

It is important to consider the heat-resistance of the 1D peanut-shaped C_60_ polymer for practical use. Nakaya et al. confirmed that the 1D polymer exhibits a heat-resistance at least up to 723 K annealing under UHV conditions [44], because the thermal heating system thus used can rise temperatures up to 723 K. This is comparable to that of polyimide film commercially available as the highest heat-resistance organic material.

In addition to the 1D peanuts-shaped C_60_ polymer described above, electronic properties of the other peanut-shaped C_60_ polymers have been investigated theoretically [45–47]. Noda et al. performed first-principles calculations of several 1D peanut-shaped C_60_ polymer models to examine their energetically stable structure and electronic properties [41]. Figure 5 shows schematic illustration of 54 different 1D peanut-shaped C_60_ polymer models optimized geometrically (T3 model was proposed by G. Wang et al. [45], and the other 53 models were derived from the T3 model *via* the GSW transformation). They consist of not only 5-/6- but also 7- and/or 8-membered rings. Unlike 1D CNTs consisting of only 6-membered ring, the 1D peanut-shaped C_60_ polymer models have both positive and negative Gaussian curvatures respectively caused by the 5- and 7/8-membered rings beside 6-membered one. It is found from the results of Figure 5 that the FP5N model is most stable energetically among the 54 models (see Table 1 of Ref. [41]). Interestingly, the energetic stability of the 1D peanut-shaped C_60_ polymers depends on their geometric components: the more octagon and pentagon-octagon pairs (heptagon and hexagon-heptagon pairs) in their structures, the more stable (unstable) the 1D polymers.

**Figure 6.**
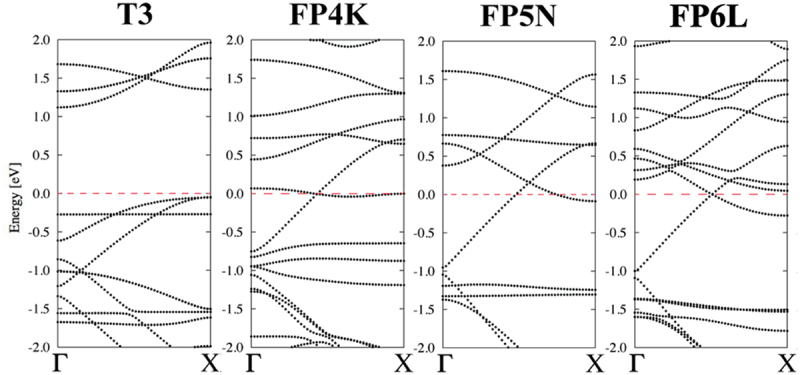
Band structures of T3, FP4K, FP5N, and FP6L polymer models shown in Figure 5. Here, the horizontal dashed red line indicates the Fermi level [41].

Reprinted with permission from Noda et al. [41]. Copyright 2015 American Chemical Society.

Figure 6 shows the band structures of typical 1D peanut-shaped C_60_ polymer models (T3, FP4K, FP5N, and FP6L) chosen among the 54 different models, and indicates that the metallic or semiconducting band structure of the 1D peanut-shaped C_60_ polymers depends on their carbon-network polygon patterns. Especially, the band structure of the FP5N model with the lowest total energy shows a metallic property (no band gap near *E*_F_).

### 2.3. Potential applications

#### 2.3.1. Ultrahigh density reversible memory atomically controlled by STM

Shrinkage in the intermolecular distances (center-to-center distance) by ca. 0.1 nm when two C_60_ molecules form a dumbbell-shaped dimer [4] is required to be detected in real space using atomic force microscope (AFM) [48] and STM [49]. This fact leads to readily discriminate C_60_ monomers and its dimers, and gives rise to utilize the dimers as a carrier of digital information representing ‘1’, whereas the monomers as that representing ‘0’. Thanks to the 1 nm size of a single C_60_ molecule, when a C_60_ dimer is used as a digital bit, it is expected that the density of digital bits reaches to more than tens of terabits per square inches ideally. This meets with the information-oriented society requiring innovative data storage technologies [50,51].

Utilizing molecular thin films as a recording media has been extensively proposed and investigated so far [52–61]. When compared to other molecules, it is noted that C_60_ molecule has following three advantages. Firstly, C_60_ molecule and its thin film are relatively stable and easy to be handled in both dry and wet processes. Secondary, well-ordered high-purity C_60_ thin films are easily produced on any substrate at RT. Thirdly, C_60_ polymerization/depolymerization in its thin film can be controlled reversibly and locally at designated positions with a nanometer precision [53,55] using STM as described below.

**Figure 7.**
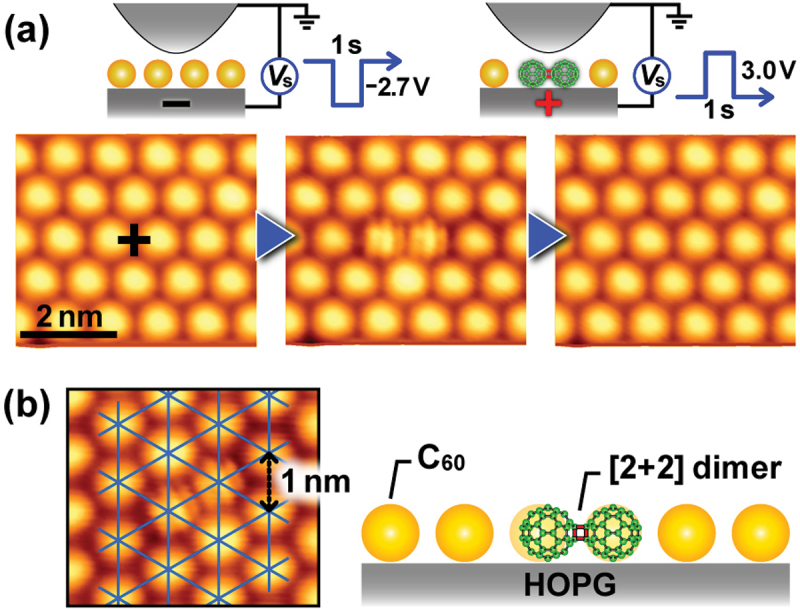
(a) High-resolution STM images showing control of polymerization (left to central) and depolymerization (central to right) between C_60_ molecules in C_60_ monolayer film using STM. (b) Local modification of molecular arrangement in C_60_ monolayer films after polymerization [65,65]. Superimposed triangular mesh in left STM image shows two-dimensional periodicity of pristine C_60_ molecules. Right picture shows schematic side view of a C_60_ dimer in the monolayer film.

To control the number of C_60_ molecules chemically bound with each other at nanoscale, local excitation is a promising way. Electronic excitation or field ionization induced by atomically sharp STM tip [62–64] has been found to control the polymerization/depolymerization area at a single C_60_ scale [53,55,60,65]. For examples, Nakayama’s group examined STM tip-induced dimerization/de-dimerization in a C_60_ monolayer on a highly oriented pyrolytic graphite (HOPG) [65]. Figure 7(a) shows a series of STM images acquired at a sample bias voltage (*V*_s_) of +1.0 V and a tunneling current (*I*_t_) of 20 pA in the same area at RT: pristine C_60_ monolayer (left), dimerization (center) induced by negative (−2.7 V) *Vs* with a pulse width of 1 s, and de-dimerization (right) induced by positive (+3.0 V) *Vs* with a pulse width of 1 s. Since C_60_ molecule is freely rotated on an inert substrate such as HOPG, STM image of the molecule shows a spherical. On the other hand, since C_60_ dimer with [2 + 2] four-membered cross-linkage is stopped rotating, STM image of the dimer shows a stripe with an internal structure [49].

Figure 7(b) shows the mesh imposed to the STM image after the dimerization (left) and schematically illustrates the corresponding dimerization (right). A slight shift in the molecular position of C_60_ dimer can be measured from the STM images [49,65].

They further examined STM images of the intermolecular bonds between C_60_ molecules in a multilayer film [53], and found that the probability of polymerization/depolymerization was higher than that in a monolayer film, because all the C_60_ molecules in a monolayer inevitably interact with the underlying substrate surface more or less. In case of a C_60_ trilayer film, the intermolecular distance in the dimer was estimated to be 0.9 nm [66], which is in good agreement with that of a dimer *via* the [2 + 2] cycloaddition reaction between C_60_ molecules [12,49,67–69]. Furthermore, it is noted that C_60_ trimers were also observed besides the dimers.

**Figure 8.**
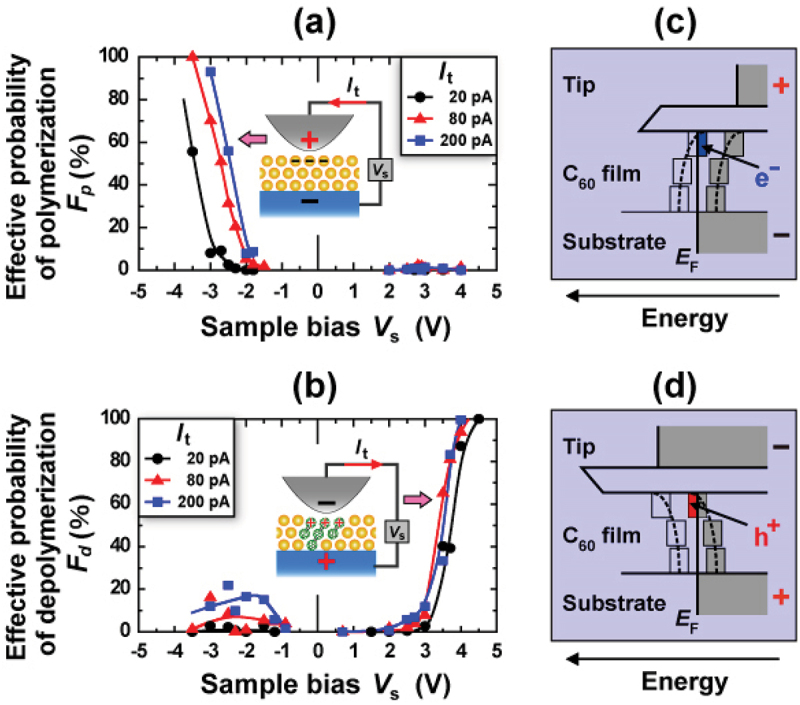
Effective probabilities of (a) Polymerization and (b) Depolymerization between C_60_ molecules induced by an STM tip in a C_60_ trilayer film [53]. The measurements were made as a function of sample bias *V*_s_ from about −4.0 to 4.5 V at different tunneling currents *I*_t_ = 20, 80, and 200 pA. Insets in (a) and (b) Schematically show that local C_60_ molecules are negatively and positively ionized due to an STM tip when negative and positive *V*_s_’s are applied, respectively. (c,d) Schematic potential energy diagrams of the whole experimental system consisting of the substrate, C_60_ trilayer film, vacuum gap, and STM tip when negative and positive *V*_s_’s were applied, respectively.

Figure 8 shows that the unbound (a) and bound (b) states of C_60_ molecules can be controlled locally and selectively just below the STM tip at RT only by changing the polarity of the *V*_s_. The high selectivity and reversible control between the monomer and polymer performed at RT is a specific feature of STM-induced C_60_ polymerization/depolymerization when compared to alternative methods such as high-temperature/high-pressure [10], photo-irradiation [70], and alkali-metal doping [11]. The reason behind such the high selectivity is due to the electric-field-induced shifts in the molecular orbital energy levels associated with charge transfer to/from substrate, namely, ionization of C_60_ molecules caused underneath the STM tip (Figure 8(c,d)). More details of the physical mechanisms of the selective induced polymerization/depolymerization have been theoretically investigated using first-principles calculations [68].

When we consider potential applications of polymerization/depolymerization control to memory devices and further to data storage technology, the density of digital bits, the speed of bit operations, and the nonvolatility of the bits play key roles in these applications. Since there have hitherto been some reports on improvement of the three key factors toward realization of polymerization/depolymerization-based data storage technologies, we will overview the status of those developments as below.

Data-storage centers are relying on the performance of magnetic hard disk drive (HDD), and it is highly demanded to increase the capacity of the data center by a higher density storage technology [53]. Tremendous work has been devoted to increase the capability of magnetic recording technology. However, since there is a certain physical limitation coming from magnetic instability when decreasing the size of a bit, a lot of studies have been carried out for future data-storage devices beyond magnetic devices so far. Kalff et al. demonstrated an example of ultrahigh density data-storage using positional operation of single chlorine atoms on Cu(100) by STM [71], and achieved a bit density of 3.2 Pbits/inch^2^ (P: peta-; × 10^15^). However, the operation of bits and keeping the bit status require ultrahigh vacuum and low-temperature conditions (bit operation was demonstrated at 1.5 K). Deoxyribonucleic acid (DNA) molecules have also been investigated by Church et al. and Erlich et al., because they have a potential to provide a huge-capacity for store data [72,73]. Although the information density of DNA-based storage device is expected to be 10^5^ times larger than that of HDD [72], essential digital operations such as DNA sequencing (encoding) and reading (decoding) is still impractical in time and costs [73].

**Figure 9.**
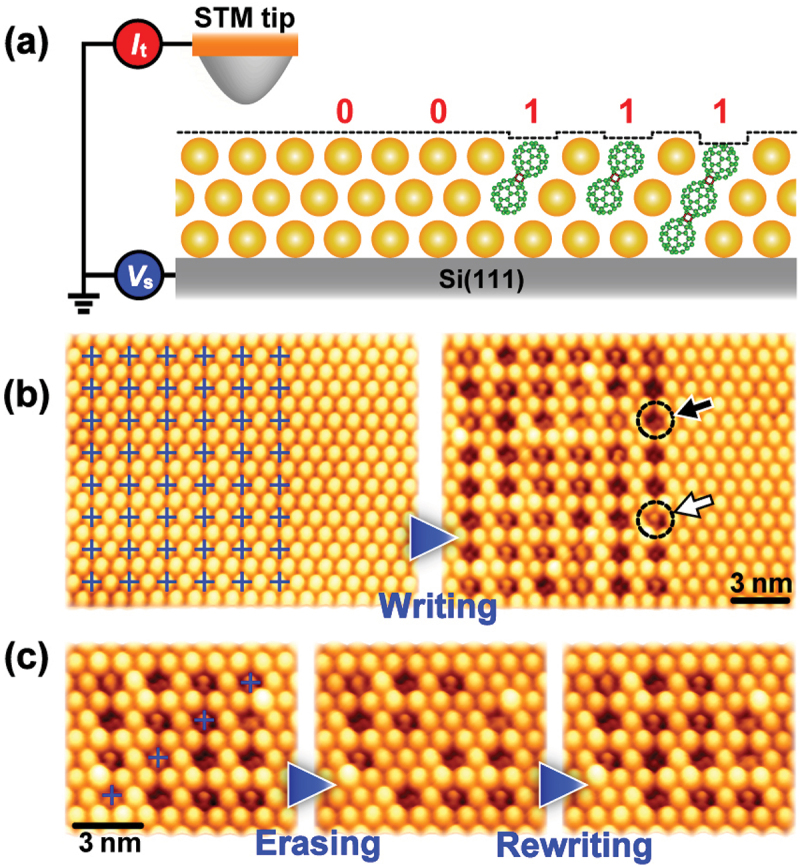
Ultrahigh-density data storage using C_60_ trilayer film as a recording media [53,58]. (a) Schematic side views of binary digital data (‘0’ and ‘1’) stored in C_60_ film. (b) STM images observed before (left) and after (right) data writing. (c) STM images showing data erasing (left to central) and rewriting (central to right). All of data storage operation were carried out at RT.

On the other hand, an ultrahigh density data storage based on local C_60_ polymerization/depolymerization has been shown to achieve 190 Tbits/inch^2^ (T: Tera-; × 10^12^) which is higher by two orders of magnitude than that of the state-of-the-art HDD devices. Figure 9 shows bit operations at a single-molecule-level using a C_60_ trilayer film at RT. The STM-induced formation of C_60_ dimers/trimers is clearly observed as a depression (dark) area (Figure 9(b)). This is because C_60_ molecules at the outermost layer react with other C_60_ molecules in the underlying layers as illustrated in Figure 9(a). These facts indicate that not only writing/erasing digital bits but also readout of the recorded bits can be performed with the same STM tip (Figure 9(c)), thus being advantageous for realizing ultra-high density molecular memory devices. In addition, the nonvolatility of the recorded bits was confirmed to be more than 1 week [53]. Although the density of digital bits using STM-induced C_60_ polymerization/depolymerization is smaller than that using atomic-scale bit operation or DNA-based data storage technology, bit operations do not require low temperatures for single chlorine atoms on Cu(100) [71] or complicated and expensive procedures for DNA encoding and decoding [73].

When the STM-induced C_60_ polymerization/depolymerization technique is practically used for high-density data storage, there are several issues to be solved. From a viewpoint of storage speed, the speed of STM-induced polymerization/depolymerization must be at late comparable to that (10^6^–10^8^ bits/sec) of the present HDD. Currently, the storage speed was reached to 3.6 × 10^2^ bits/s at fast [58], which is actually limited by a pulse width (a few ms) of the applied voltage to induce C_60_ polymerization/depolymerization. Further study is necessary to achieve data writing and erasing with a speed of ca. 10^6^ bits/s for practical use by improving the quantum efficiency (*η*) of both polymerization (*η*_p_ = 2 × 10^−7^ reaction/hole) and depolymerization (*η*_d_ = 3 × 10^−8^ reaction/electron).

#### 2.3.2. CO_2_ immobilization

Immobilization and reuse of environmentally hazardous compounds such as CO_2_, NO_*x*_, or SO_*x*_ will play key roles in solving our environmental and energy issues [74,75]. One possible way is to use porous materials with nanospace [76]. Given that these nanoporous materials exhibit high electrical conductivity or high heat-resistance, electrochemical or heat treatments can be utilized to enhance chemical reactions in their nanospace.

As described in Sections *2.1* and *2.2*, the 1D peanut-shaped C_60_ polymer film has a HCP structure shown in Figure 3, which results in formation of sub-nm spaces with a size of ca. 0.3 nm and 0.6 nm periodically arranged (Figure 1 of Ref. [76]). In addition, the 1D polymer exhibits physical properties arising from 1D metal and a high heat-resistance at least up to 723 K. These findings suggest that the robust sub-nm space can act as a specific reaction field in a similar manner to those of conventional porous materials [74–81].

After the 1D peanut-shaped C_60_ polymer film was exposed to atmospheric air for 30 min at RT, Nakaya et al. examined the change in *in situ* infrared (IR) and mass spectra of the 1D C_60_ polymer under UHV conditions in combination with first-principles calculations based on DFT when compared to those of the pristine polymer (Figure 3 and 4 of Ref. [82]), and found that CO_2_ is immobilized as a carbonate ion (CO_3_^2–^) in the sub-nm space of the 1D polymer film *via* [CO_2_ + H_2_O] reaction [51], despite the fact that the reaction [CO_2_ + H_2_O] is hard to proceed at RT in the gas phase (the activation energy, *E*_a_ = ca. 2 eV [83]). Although a pristine C_60_ film also has a similar sub-nm space inside, it was confirmed that no significant differences in their IR spectral features before and after atmospheric-air exposure are observed (Figure S2 in the supporting information of Ref. [82]). Thus, the 1D metallic peanut-shaped C_60_ polymer as a framework of the sub-nm space also plays a crucial role in promoting the reaction at RT.

**Figure 10.**
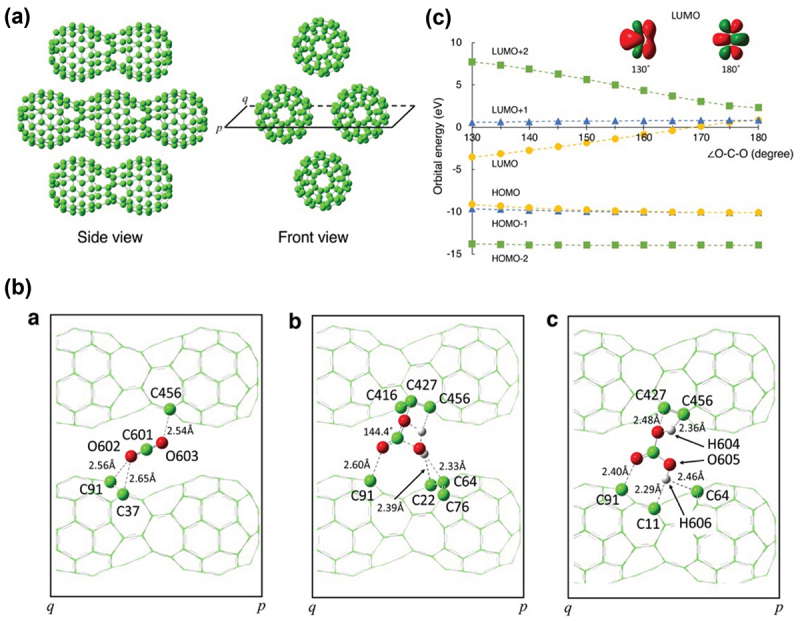
(a) Model structure of 1D peanuts-shaped C_60_ polymer film for quantum chemical calculations. (B) Optimized molecular structure of (a) CO_2_, (b) transition state (TS) obtained at a lower convergence, and (c) H_2_CO_3_ in the sub-nm space. Symbols *p* and *q* correspond to those in the front views of Figure 5(a). The contacting carbon atoms and adsorbed molecules are shown as ball-models, and are connected by dashed lines together with their distances. (C) Change in the frontier orbital energies of CO_2_ with respect to ∠O–C–O bond angle. At each angle, only the C–O bond distance was optimized. As the angle decreases, only the LUMO energy decreases monotonically and becomes negative below 170°, implying that the LUMO has an electron affinity [82].

To reveal the mechanisms behind the reaction occurred at RT in the sub-nm space, Kitagawa’s group theoretically examined the most energetically preferable location of CO_2_ molecule in the sub-nm space, using a sub-nm space model (Figure 10(a)). The electronic structure of the model obtained at the PM6 level of theory exhibited a weak charge polarization especially around the bridged concave portions (Figure S3a of Ref. [82]). Figure 10(b) shows the optimized geometrical structure of the CO_2_–H_2_O transition state (TS) in the sub-nm space, whereas Figure 10(b) shows that of H_2_CO_3_ formed from the reaction. Only under a lower convergence condition, one TS was found along the reaction coordinate (Figure S4 in the supporting information of Ref. [82]), whereas they found product H_2_CO_3_ directly *via* no TSs under a higher convergence condition (Figure 10(b)). This suggests that the *E*_a_ of [CO_2_ + H_2_O] reaction is very small or negligible in the sub-nm space of the 1D polymer film, which resembles a proton transfer process [84]. Figure 10(c) shows the dependence of valence molecular orbital (MO) energies on the ∠O-C-O bond angle. The lowest unoccupied MO (LUMO) energy was lowered monotonically to be a negative value at bond angles below 170°. This implies that the LUMO has an electron affinity. Furthermore, the insets show that a carbon pσ-type orbital of the LUMO expands more toward the obtuse-angle side of CO_2_ at smaller ∠O-C-O angles. This suggests that the bending motion promotes [CO_2_ + H_2_O] reaction from the obtuse-angle side, giving rise to a decrease in the *E*_a_. Thus, factors such those mentioned above work together to promote the reaction at RT *via* no or negligible TSs. Alternative mechanisms were also discussed, but they can be excluded (Supporting information in Ref. [82]).

Nakaya et al. [82]; licensed under a Creative Commons Attribution (CC BY) license.

There have been many conventional methods to activate CO_2_ radioactive-chemically, thermochemically, biochemically, photochemically, electrochemically, and catalytically [85]. The common idea among them is to add electrons to LUMO of CO_2_, because the LUMO features an antibonding orbital. Thus, the reduction of CO_2_ makes O=C=O bonding weakened to become reactive. On the other hand, in the nanospace of 1D peanut-shaped C_60_ polymer film, CO_2_ is activated at RT *via* enhancement in its angular vibration by CO_2_ molecule pinning due to local Coulomb interactions, which reduces the LUMO energy to be negative (electron affinity emerges).

The CO_2_ uptake performance of the 1D C_60_ polymer film is estimated to be 1.60 mmol/g, which is comparable to that of zeolites such as Li-LSX (1.34 mmol/g) and Na-LSX (0.87 mmol/g) obtained under the similar exposure conditions (in 1 atm air at RT) [86].

#### 2.3.3. 1D N-doped peanut-shaped C_60_ polymer

To mitigate the greenhouse effects, considerable efforts have been made to convert CO_2_ to valuable industrial chemicals. The widely used catalysts for CO_2_ reduction are metals [87], metal oxides [88], metal chalcogenides [89] and metal – organic complexes [90]. However, these metal-containing catalysts are costly and their energy efficiency is limited. Peanut-shaped carbon nanotubes (PSNTs) are of particular interest because of their exotic geometry with a large specific surface area and with positive and negative Gaussian curvatures respectively with negative and positive polarizations. Thus, PSNTs are expected to have good performance for CO_2_ capture and conversion [91,92].

**Figure 11.**
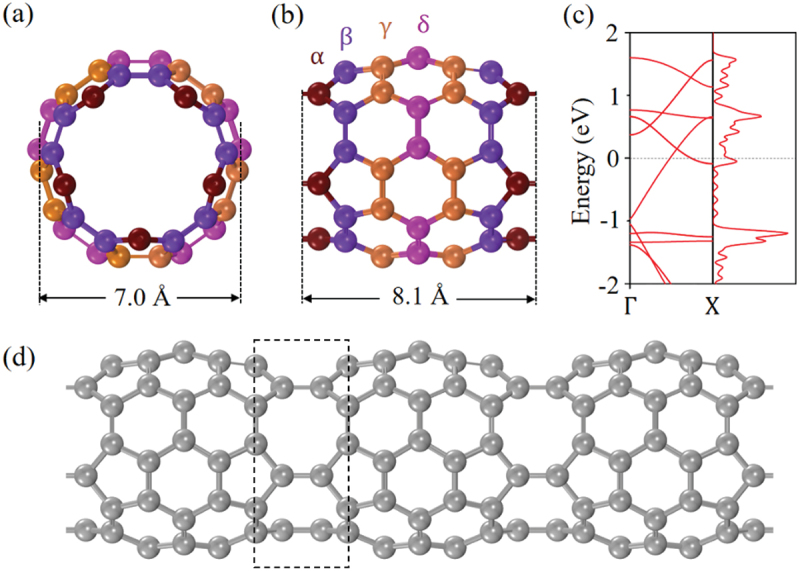
(a) Front and (b) Side view of FP5N. (c) Electronic band structure and DOS of FP5N. (d) Side view of a 1×1×3 supercell of FP5N [92].

Reprinted with permission from Zhou et al. [92]. Copyright 2019 Elsevier.

As shown in Figure 5, there are many types of 1D PSNTs studied theoretically, and FP5N was found to be energetically stable with a high symmetry and to exhibit a metallic feature [41], which share the structural features of C_60_ cages and 1D uneven peanut-shaped structures. Figure 11 shows the geometric structure of front (a) and side (b, d) views for FP5N, along with the electronic band structure and DOS (c). There exist 5-, 6- and 8-membered rings where the C atoms have much more complicated bonding environments that offer more flexibility to tune the reactivity when compared to conventional 1D CNTs. This enables us to improve the catalytic performance. Figure 11(c) shows that FP5N is metallic as the partially occupied bands cross the *E*_F_. When one nitrogen (N) atom is substitutionally doped into the C-framework of FP5N, there are four chemically nonequivalent doping sites (α, β, γ, and δ shown in Figure 11(b)).

The total energy calculations for the four N-doped FP5N configurations were carried out to determine the most preferred doping site. The configuration with N at γ site was found to be most stable, which is lower in energy by 0.04, 0.07 and 0.17 eV than the configurations with N at α, β and δ sites, respectively. Then, Wang et al. calculated the band structure of the most stable structure of N-doped FP5N.

**Figure 12.**
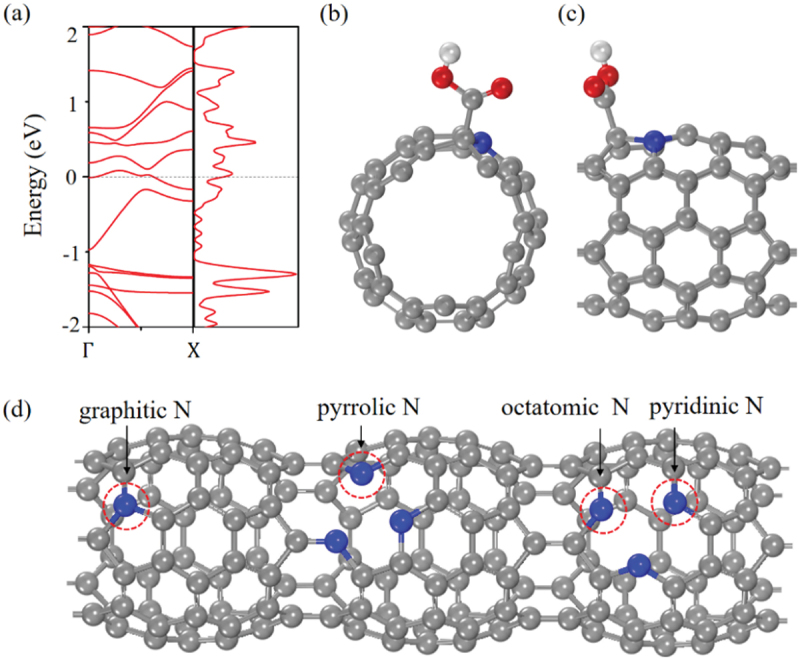
(a) Electronic band structure and DOS of N-doped FP5N structure. (b) Front and (c) Side view of •COOH adsorbed on N-doped FP5N. (d) Schematic diagram of different N doping catalytic sites on FP5N [92].

Reprinted with permission from Zhou et al. [92]. Copyright 2019 Elsevier.

Figure 12(a) reveals that the metallic feature still remains after N doping at *γ* site. When more N atoms are substitutionally doped into FP5N, the possible N doping sites were categorized into three different types: pyridinic N (in the hexagonal ring), pyrrolic N (in the pentagonal ring), and octatomic N (in the octatomic ring), as shown in Figure 12(d). Each of these sites is unique in the N-doped FP5N structure. The preferred electrocatalyst candidates were screened by calculating the free energy changes for both •COOH and •OCHO radicals at all the catalytic sites in the first proton-coupled electron transfer (PCET) step [93]. The free energy change of •COOH adsorption was found to be much smaller than that of •OCHO. This suggests that the first intermediate PCET step for CO_2_ electrocatalytic reduction (CO_2_ER) is •COOH. The geometrical structure of •COOH adsorbed on top of the C atom near the graphitic N is shown in Figure 12(b) (front view) and (c) (side view).

**Figure 13.**
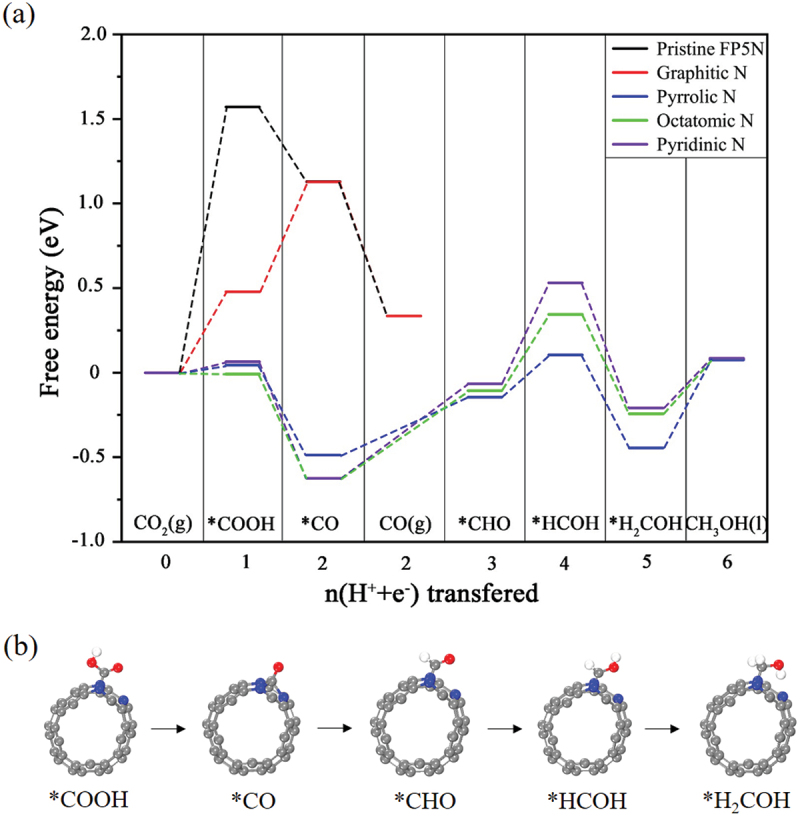
(a) Free energy diagrams of different catalytic sites for pristine and N-doped FP5Ns. (b) Configurations for the intermediates of •COOH, •CO, •CHO, •HCOH, and •H_2_COH for CO_2_ER on the octatomic N [92].

Reprinted with permission from Zhou et al. [92]. Copyright 2019 Elsevier.

Figure 13 shows (a) the free energy diagram for the lowest energy pathway of CO_2_ converted to CO or CH_3_OH by including the zero-point energy, vibrational entropy and solvent correction, and (b) the corresponding intermediate configurations. It is found that the pristine FP5N has the highest free energy barrier for the first step of CO_2_ adsorption, which hinders CO_2_ conversion. The potential-limiting step [•COOH → •CO] for graphitic N is 0.65 eV, and •CO prefers to decompose to CO rather than getting further hydrogenated because of the much more negative free energy change of [•CO → CO (g)] reaction. Even though the energy change (0.65 eV) for the graphitic N is larger than that of the pyrrolic N (0.52 eV), pyridinic N (0.52 eV) and octatomic N (0.60 eV), it still lowers the overpotential by 0.92 V as compared to that in the pristine FP5N, which produces CO at a higher overpotential of 1.57 V. This significantly improves CO_2_ conversion performance by N doping. Furthermore, since the binding energy of •COOH to the graphitic N (2.67 eV) is higher than that of the pristine FP5N (1.58 eV), •COOH binds more strongly on the graphitic N.

During the CO_2_ conversion, the first electron transferred to the chemically stable CO_2_ usually possesses a high overpotential. On the contrary, in the case of FP5N, the free energy changes of [CO_2_ → •COOH] reaction for pyrrolic N, octatomic N and pyridinic N are estimated to be only 0.04, −0.01 and 0.06 eV, respectively. These free energy changes are rather small in contrast to the pristine FP5N (1.57 eV), the graphitic N (0.48 eV) and many more promising electrocatalysts including transition metal Cu (0.41 eV). The binding energies of •COOH to pyrrolic N, octatomic N and pyridinic N are estimated to be 3.12, 3.17 and 3.10 eV, respectively, which are all higher than that of graphitic N. This is because pyrrolic N, pyridinic N and octatomic N are electron-rich, which bind CO_2_ while electrons of graphitic N are located in the π* antibonding orbital, making them less accessible for CO_2_ binding. The strong binding of CO_2_ to pyrrolic N, pyridinic N and octatomic N sites accounts for the small free energy change for the first step: [CO_2_ → •COOH]. The free energy changes of [•CO → CO (g)] reaction at pyrrolic N, octatomic N and pyridinic N are all larger than that of [•CO → •CHO] reaction. This indicates that •CO is likely to produce •CHO rather than CO gas and further hydrogenated.

The potential-limiting steps for pyrrolic N, octatomic N, and pyridinic N are found to be [•H_2_COH → CH_3_OH (g)], [•CHO → •HCOH], and [•CO → •CHO] reactions, respectively. The corresponding overpotentials of CO_2_ conversion are estimated to be 0.52, 0.52 and 0.60 V, respectively, which makes the whole reaction being spontaneous and exergonic. For octatomic N, the CO_2_ conversion pathways are identified to be as shown in Figure 13(b) (front view).

$$\mathrm{CO}2\left(g\right)\to ∙\mathrm{COOH}\to ∙\mathrm{CO}\to ∙\mathrm{CHO}\to ∙\mathrm{HCOH}\to ∙H2\mathrm{COH}\to \mathrm{CH}3\mathrm{OH}\left(l\right),$$

Since the large free energy change of [•CO → CO (g)] reaction makes •CO further hydrogenated, the final product is CH_3_OH for pyrrolic N, octatomic N and pyridinic N. This is rare for all kinds of N-doped carbon nanomaterials as catalysts for CO_2_ conversion. When compared to metal catalysts, the N-doped PSNTs also have some merits. For instance, the overpotential of Mo-Bi bimetallic chalcogenide for reducing CO_2_ into CH_3_OH is 0.7 V [94], and among the metal electrocatalysts studied previously, copper is the only metal that is capable of reducing CO_2_ to significant amounts of hydrocarbons and oxygenates with high overpotentials (0.9–1.1 V) [95,96], which is higher than that of N-doped PSNT. Thus, this finding demonstrates that the N-doped PSNT is a promising candidate to replace metal catalysts for effective CO_2_ reduction.

In addition, it is also important to note that PSNTs possess much lower lattice thermal conductivity as compared to conventional carbon nanotubes (CNTs) besides the high performance for CO_2_ immobilization and conversion. By using non-equilibrium molecular dynamics simulations and lattice dynamics together with DFT, Sun et al. found that the thermal conductivity of the PSNT is reduced by more than 90% as compared to that of CNTs and remains almost the same when different strains applied [97,98]. This exhibits very different behaviors from those of CNTs (the thermal conductivity decreases monotonically with increasing strain). The insensitive response of thermal conductivity against strain is due to the insensitivity of its phonon DOS and group velocity against strain. Furthermore, when 5–8 membered defect is introduced to PSNT, the lattice thermal conductivity of PSNT with the defect is less by one-tenth than that of pristine CNT with a similar radius. This arises from the low phonon group velocity, short relaxation time, large lattice vibrational mismatch and strong anharmonicity [97]. These findings provide new insight for 1D PSNT going beyond conventional CNTs.

## 3. Two-dimensional dumbbell-shaped C_60_ polymers and related nanocarbons formed *via* photo-induced polymerization

### 3.1. Structures

Since the first report on C_60_ polymerization by Ar-ion laser or UV-vis lamp irradiation of a pristine C_60_ film at RT [70] by Rao et al., Onoe et al. investigated the structure of photopolymerized C_60_ films using *in situ* FT-IR spectroscopy [16], *in situ* x-ray photoelectron spectroscopy (XPS) [17,18], and *in situ* STM [20,49] under UHV conditions. The results of FT-IR, XPS, and STM found that C_60_ molecules are polymerized to form 1D/2D C_60_ polymer with a cross-linkage of [2 + 2] cycloadditional four-membered ring.

**Figure 14.**
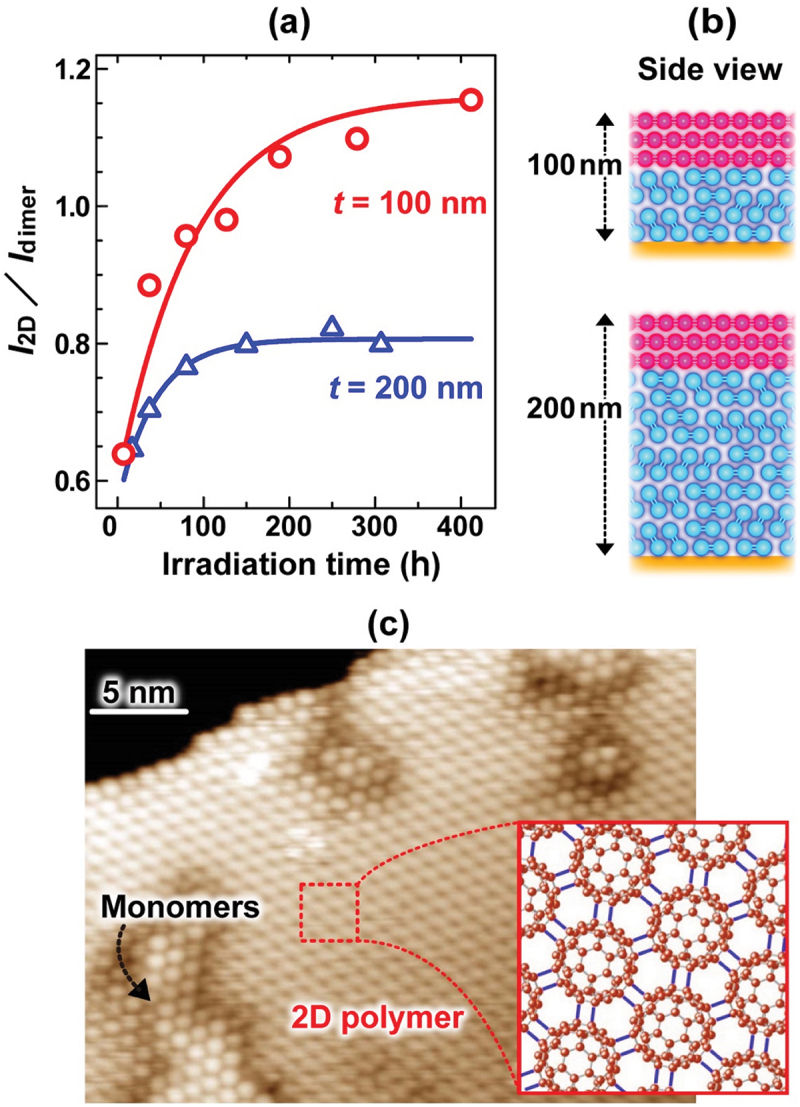
(a) The dependence of the intensity ratio (*I*_2D_/*I*_d_) on photo-irradiation time for 100 nm- (red) and 200 nm-thick (black) C_60_ films on CsI, (b) Schematic model for the distribution of the dimer (blue) and 2D polymer (red) in each film, and (c) STM image of 100-h photo- irradiated C_60_ multi-layers formed on Si(111)/√3×√3-ag substrate [20].

Figure 14 shows (a) the dependence of the IR intensity ratio (*I*_2D_/*I*_dimer_) on photo-irradiation time for 100 nm- (red) and 200 nm-thick (black) C_60_ films on cesium iodide (CsI) substrates, (b) schematic model for the spatial distribution of the dimer (blue) and 2D polymer (red) in each film, and (c) STM image of 100-h photo-irradiated C_60_ thin film formed on the Si(111)/∏3×∏3-Ag substrate. Figure 14(a) shows that the IR intensity ratio of the 200 nm-thick C_60_ film (blue) increased remarkably until 100-h irradiation and became saturated after 150-h irradiation, whereas that of the 100 nm-thick C_60_ film (red) increased remarkably until 200-h irradiation and is extrapolated to be saturated after 500-h irradiation. It is found that the saturated (*I*_2D_/*I*_dimer_) value of ca. 1.15 for the 100 nm-thick film was larger than that of ca. 0.8 for the 200 nm-thick film. This difference can be well explained by assuming that the 2D dumbbell-shaped C_60_ polymer is formed within a few surface layers, as shown in Figure 14(b). This assumption is strongly supported by the results obtained using XPS (photoelectrons were measured from a few nm in depth of the film) [17,18]. In addition, it was observed from the result in Figure 14(c) that the 2D [2 + 2] dumbbell-shaped C_60_ polymer is formed on the outermost C_60_ layer. Furthermore, even if the film thickness increases, the formation region of the 2D polymer from the outermost layer should remain unchanged as long as the same photo-irradiation time. Thus, the saturated (*I*_2D_/*I*_dimer_) value of the 100 nm-thick film was greater than that of the 200 nm-thick one.

Onoe et al. [20]; licensed under a Creative Commons Attribution (CC BY) license.

For practical use of the 2D dumbbell-shaped C_60_ polymer, it is important to consider its heat resistance. Unlike the 1D peanut-shaped C_60_ polymer film, the [2 + 2] four-membered ring was drastically dissociated at temperatures exceeding 373 K with an activation energy of 1.25 eV [69]. Accordingly, the 2D C_60_ polymer is limited to be used for devices that not only work below 373 K but also are fabricated in all processes below the temperature.

More recently, semiconducting monolayer 2D C_60_ networks (band gap: ca. 1.6 eV) *via* cross-linking of C–C single and [2 + 2] cycloaddition bonds have been reported to be synthesized by Hou et al. and indicated no thermal decomposition after heating at 600 K for 10 min [99]. However, since the decomposition of the 2D polymer proceeds on the basis of the kinetics with some activation energy as heating time becomes long, the heating condition thus examined is too short to confirm the heat resistance of the 2D monolayer C_60_ networks. Accordingly, it is further necessary to investigate the heating-time dependence of the 2D network decomposition. Section 3.3.2 will discuss the kinetics of thermal decomposition of a photopolymerized C_60_ film with [2 + 2] cycloaddition bonds.

### 3.2. Fundamental properties

Onoe and Nakayama et al. examined the electrical properties of the 2D C_60_ photopolymer using four-probe measurement [19]. Although Okada and Saito theoretically predicted the 2D polymer to be a semiconductor with a band gap smaller by one-third [100] than that (1.85 ± 0.04 eV) for pristine C_60_ solids [101], no attempts to examine the electrical properties of the 2D C_60_ photopolymer have been made until then.

**Figure 15.**
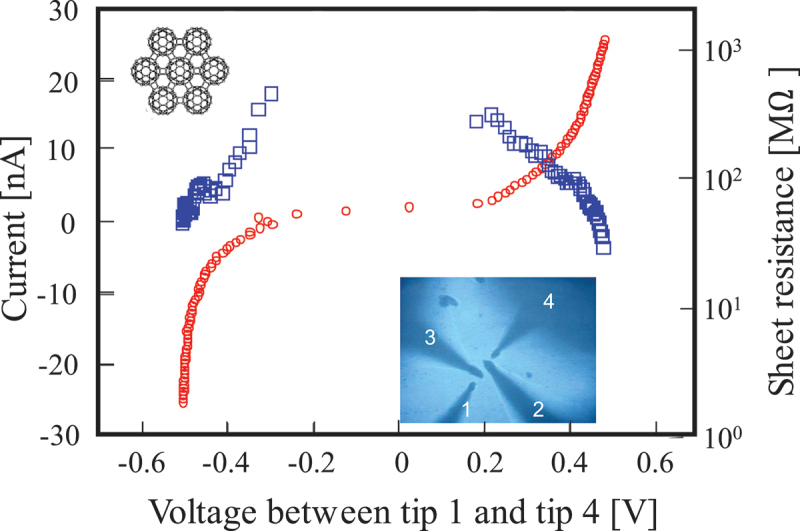
Sheet resistance (blue square) and current – voltage curve (red circle) of the photopolymerized C_60_ film using four-probe measurements in air at RT. Inset shows the optical micrographs obtained during the measurements. A typical distance between adjacent probes was set in the range of 8–30 μm [19].

Figure 15 shows the *I–V* characteristics and sheet resistance of photopolymerized C_60_ film (after 400 h UV-vis irradiation) obtained in atmospheric air at RT. Here, the red circles represent the *I–V* characteristics measured using probes 1 and 4 (outer probes), whereas the blue squares represent the sheet resistance derived from voltages measured by probes 2 and 3 (inner probes), as shown in the inset. The sheet resistance and *I–V* characteristics of a pristine C_60_ film could not be obtained, because its sheet resistance was beyond the range of the measurement system at that time. This was presumably due to oxygen in air which is well known to act as an electron capture (O_2_ is well known to become O_2_^–^ as a superoxide radical). Judging from the inset of the optical micrograph taken during the corresponding four-probe measurements, the 2D dumbbell-shaped C_60_ polymer film seems to be uniformly smooth and to have no grain boundaries within the measurement area. The typical inter-probe distance was set in the range of 8–40 μm, ensuring an appropriate film thickness for the four-probe measurements. When the inter-probe distance (*L* = 8–40 μm) is much larger than the film thickness (*t* = 70 nm), the sheet resistance (*R*_s_) of the film can be evaluated in terms of *R*_s_ = 4.532 × (*V/I*), and the resistivity (*ρ*) can be calculated from *ρ* = *R*_s_ × *t*. Here, *V* is the difference in voltage between the two inner probes, and *I* is the current flow between the two outer probes.

Onoe et al. [19]; licensed under a Creative Commons Attribution (CC BY) license.

As shown in Figure 15, the *I–V* curve demonstrates that the 2D C_60_ polymer is a non-doped semiconductor (except during the plateau of the *I – V* curve). Since the *R*_s_ depends on the current flow between the two outer probes, the resistance thus obtained does not correspond to the carrier density of the 2D C_60_ polymer film. The carriers in the film are apparently supplied by the biased metal probe during the measurements. Namely, the 2D C_60_ polymer has no carriers (electrons or holes) contributing to the conductivity at RT. This makes it difficult to estimate the resistivity of the polymer using the four-probe measurement system at that time. In addition, although the temperature dependence of *R*_s_ should be measured for the 2D C_60_ polymer in order to determine the energy gap of the film from Arrhenius plot, the experimental system resulted in temperature variations only from RT to ca. 373 K, which were insufficient to estimate the energy gap. However, considering that *R*_s_ and *I–V* characteristics of the pristine C_60_ film could not be obtained using the measurement system, these results nonetheless demonstrate that the 2D photopolymer is a semiconductor, which is consistent with the theoretical prediction [100]. According to first-principles calculations of C_120_ dumbbell-shaped dimer (Figure 4 of Ref. [19]), it is clear that nodes where no electrons exist are formed at the [2 + 2] four-membered ring between adjacent C_60_ molecules for the HOMO. This indicates that electrons cannot move from one C_60_ to another without excitation-induced hopping. In other words, there is some energy gap for the electron hopping between adjacent C_60_ molecules *via* the ring. Consequently, the 2D dumbbell-shaped C_60_ polymer has a semiconducting property. On the other hand, electrons can move over the whole dumbbell dimer for the LUMO, indicating that the 2D C_60_ polymer exhibits conducting properties when electrons are excited from the HOMO to the LUMO.

For a further study, we will examine the temperature dependence of *R*_s_ for the 2D C_60_ polymer film using *in situ* UHV four-probe measurement apparatus equipped with a helium cryostat [102,103].

### 3.3. Potential applications

#### 3.3.1. Organic solar cells

Since C_60_ has a large electron affinity of 2.68 eV [104], C_60_ is often used an electron acceptor for organic solar cells (OSCs) that expects to be one of the wearable power generators. After photogenerated excitons are separated to electron/hole carriers at a donor/acceptor interface, the electrons diffuse to a cathode through the C_60_ film. However, since the resistivity of pristine C_60_ film is very high (2 × 10^6^ Ωcm [43]) even under UHV conditions, it is one of the main factors to lower the energy conversion efficiency of OSCs. As described in the previous section, photopolymerized C_60_ film exhibits a semiconducting property with the LUMO where electrons can move from one to another *via* the [2 + 2] bond between adjacent C_60_ molecules (Figure 4 of Ref. [19]).

**Figure 16.**
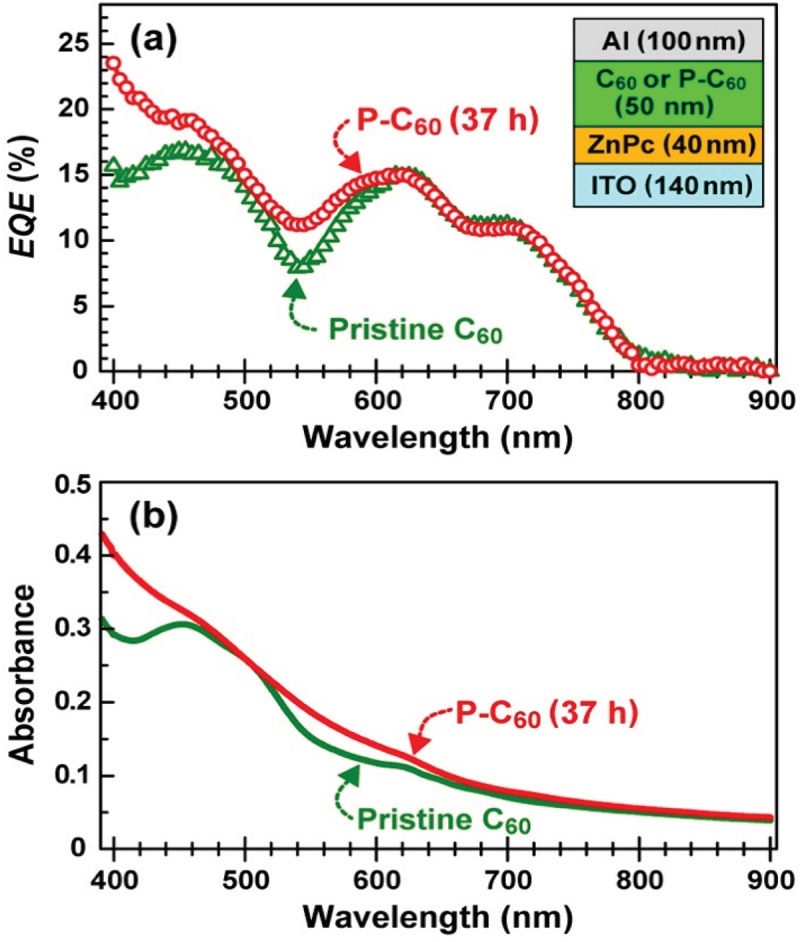
(a) *EQE* spectra of [ITO (140 nm thick)/ZnPc (40 nm thick)/C_60_ (50 nm thick)/Al (100 nm thick)] (green), and [[ITO (140 nm thick)/ZnPc (40 nm thick)/P-C_60_ (50 nm thick)/Al (100 nm thick)] (red) OPV cells, and (b) UV-vis-NIR spectra of pristine (green) and photopolymerized (red) C_60_ films in a wavelength region of 400–900 nm [13].

From this standpoint, Kato et al. [13] investigated the modification of the structural and optical properties of C_60_ film used as an acceptor in terms of photopolymerization between adjacent C_60_ molecules in order to improve the external quantum efficiency (*EQE*) of Zinc phthalocyanine (ZnPc)/C_60_ OSC. Figure 16 shows (a) the *EQE* of [ITO/ZnPc/P-C_60_/Al] (red) and [ITO/ZnPc/C_60_/Al] (green) OPV cells (Inset schematically illustrates the OPV cell structures), and (b) UV-vis-NIR spectra in a wavelength region of 400– 900 nm. As shown in Figure 16(a), the *EQE* increased in a wavelength region both of 400–480 nm and of 520–580 nm for photopolymerized C_60_ film (named as p-C_60_ film) used instead of the pristine one, which well corresponds to the increase in the absorbance of p-C_60_ film in both the wavelength regions as shown in Figure 16(b). In addition, since the structure of ZnPc film used as a donor remained unchanged after 37-h photo-irradiation (Figure 3 of Ref. [13]), its optical characteristic is considered to be unchanged. In fact, they confirmed no changes in UV-vis-NIR spectra of 40 nm-thick ZnPc film even after 74-h UV-vis photo-irradiation (photon energy: 2–4 eV, fluence: 0.4 W cm^−2^), as shown in Figure S1 of Ref. [11]. Consequently, the increase in *EQE* in those wavelength regions is mainly due to an increase in the number of photogenerated intra- and inter-molecular excitons in p-C_60_ film [105,106]. Details of discussion on the other structural and physical properties of C_60_ film influenced by photo-irradiation have been described in Ref. [11].

Kato et al. [13]; licensed under a Creative Commons Attribution (CC BY) license.

#### 3.3.2. Organic thermoelectric materials

C_60_ films exhibit a giant Seebeck coefficient (*S*) of more than 150 mV K^−1^ (at 300 K) [107] and a lower thermal conductivity (*k*) of 0.4 W m^−1^ K^−1^ (at 300 K) [108] when compared to those (*S* = 0.27 mV K^−1^, *k* = 1.6 W m^−1^ K^−1^ at 300 K) of Bi_2_Te_3_ commercially available [109], thus being expected to be one of powerful candidates for next-generation wearable power generator used to wearable devices (sensors, etc.). However, the electrical conductivity (σ) of C_60_ films was reported to be an order of 10^−5^ Ω^−1^ cm^−1^ [44], which is quite smaller than that (307 Ω^−1^ cm^−1^) of Bi_2_Te_3_ [109]. According to the figure of merits (*Z = S*^*2*^*σk*^−1^) used as a performance index, it is necessary to increase the σ of C_60_ films drastically while suppressing the decrease in the *S* for practical use, because there is the trade-off relationship between *S* and σ (the Mott’s formula) [110]. For instance, a typical way to increase the σ of C_60_ films is to use dopants such as alkali metals (potassium: K) [44,111] and organic di-metal complexes [112] into the films. However, while the σ was increased with a fraction of the complexes in the film, the *S* was comparably decreased.

One possible way to solve this issue is to utilize C_60_ photopolymerization, because a part of polymerization in C_60_ films contribute to increase the σ while suppressing the decrease in the *S*. Actually, the polymerization reduces their intermolecular distance by ca. 10% *via* the [2 + 2] four-membered ring, thus increasing the σ [19,20]. This idea is quite similar to that of the carrier energy filtering effects [113,114]. This inversely consider that a perfect C_60_ polymer network *via* the [2 + 2] four-membered ring is randomly broken to suppress a phonon-mediated thermal conductivity. When C_60_ molecules are polymerized, the absorption band of C_60_ films correspondingly becomes broadened toward a longer wavelength [13]. Accordingly, the modification of the electrical and optical properties by photopolymerization is useful to improve the device performance such as OSCs, as shown in Figure 16.

Thus, the control of the proportions of individual C_60_ photopolymers is expected to further modify the physicochemical properties of C_60_ films for the device performance. To the best of our knowledge, there have been no reports to discuss the proportion distribution for C_60_ monomer, dimer, trimer and larger oligomers kinetically so far, though Metelov et al. reported on depolymerization of C_60_ dimer [115] and two-dimensional (2D) rhombohedral C_60_ polymer [116] synthesized by the HPHT method.

**Figure 17.**
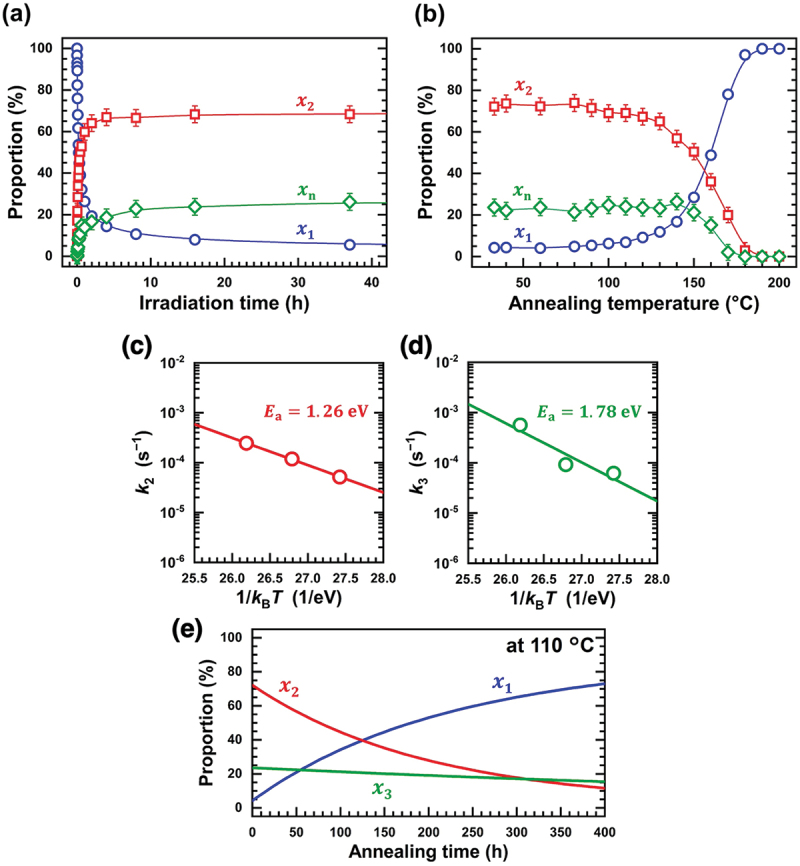
(a) Irradiation-time dependence of individual proportions of C_60_ monomer (*x*_1_: blue), dimer (*x*_2_: red), and trimers/oligomers (*x*_n_: green), (b) Annealing-temperature dependence of *x*_1_ (blue), *x*_2_ (red), and *x*_n_ (green), (c, d) The Arrhenius plots for *k*_2_ and *k*_3_, and (e) The annealing-time dependence of *x*_1_ (blue), *x*_2_ (red), and *x*_3_ (green) at 110°C [117].

Izumi et al. studied the kinetics of photopolymerization and depolymerization in C_60_ films using *in situ* Fourier-transform infrared spectroscopy for the development of C_60_-based thermoelectric materials [117]. Figure 17 shows (a) the irradiation-time dependence of individual proportions of C_60_ monomer (*x*_1_: blue), (C_60_)_2_ dimer (*x*_2_: red), and (C_60_)_n_ (n ≧ 3) oligomers (*x*_n_: green), (b) the annealing-temperature dependence of those proportions, (c, d) the Arrhenius plots for rate constants *k*_2_ [(C_60_)_2_ → 2 C_60_] and *k*_3_ [(C_60_)_3_ → (C_60_)_2_ + C_60_], and (e) the annealing-time dependence of *x*_1_ (blue), *x*_2_ (red), and *x*_3_ (green) at 110°C [117]. As shown in Figure 17(a), the proportion of the monomer (*x*_1_), dimer (*x*_2_), and oligomers (*x*_3_) was saturated to be 4%, 72%, and 24%, respectively, after 40 h irradiation of ultraviolet light (fluence: 0.2 W cm^−2^, wavelength: 300–410 nm). This indicates that the polymerization between adjacent (C_60_)_2_ dimers is very difficult to occur. In other words, to increase the proportions of (C_60_)_n_ oligomers, [C_60_ + (C_60_)_n-1_] reaction should be proceeded.

Izumi et al. [117]; licensed under a Creative Commons Attribution (CC BY) license.

**Figure 18.**
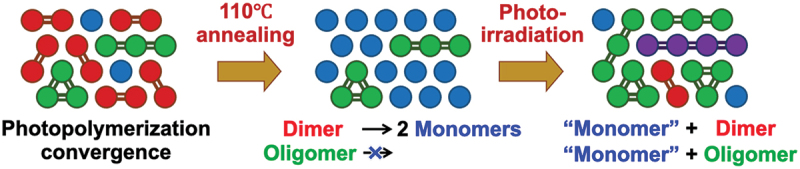
Schematic illustration for controlling the proportion of (C_60_)_n_ oligomers *via* the iterations of [photopolymerization/110°C-thermal decomposition] cycle.

They further examined the decomposition kinetics, and it is interesting to note from Figure 17(b) that *x*_2_ (the dimer) decreases at temperatures exceeding 100°C, whereas *x*_3_ (the oligomers) remains constant up to 140°C. Correspondingly, the *E*_a_ of decomposition for (C_60_)_2_ dimer and (C_60_)_n_ (n ≧ 3) oligomers was obtained to be 1.26 eV and 1.78 eV from the Arrhenius plots of *k*_2_ (c) and *k*_3_ (d), respectively. Details of discussion on the reason behind the *E*_a_ of the trimers larger than that of the dimer have been described in Ref. [117]. Accordingly, one given annealing temperature in the range of 100–140°C makes it possible to proceed the decomposition of (C_60_)_2_ dimer with remaining the proportion of (C_60_)_n_ (n ≧ 3) oligomers unchanged. Figure 17(e) shows the results of *x*_1_, *x*_2_, and *x*_3_ at an annealing temperature of 110°C. The proportion of the (C_60_)_2_ dimer (*x*_2_) becomes smaller than that of the (C_60_)_3_ trimers (*x*_3_) after more than 300 h. Thereafter, further photoirradiation will induce the polymerization of [C_60_ + (C_60_)_n-1_ (*n* > 3) → (C_60_)_n_] to increase the *x*_n_ value. Therefore, the iterations of [photopolymerization/110°C-thermal decomposition] cycle can control the proportion of (C_60_)_n_ oligomers to modify the physicochemical properties of photopolymerized C_60_ films for application to C_60_-based thermoelectric materials, as shown in Figure 18.

Actually, Nakaya et al. obtained that the power factor (*PF* = *S*^2^σ), which is one of the important indexes for thermoelectric device performance, of C_60_ film after 8 h UV-vis photoirradiation (*PF* = 2.3 × 10^−5^ W m^−1^ K^−2^) became greater by one order than that (2.3 × 10^−6^ W m^−1^ K^−2^) of pristine C_60_ film [118]. Considering that *PF* of more than 10^−3^ W m^−1^ K^−2^ is enough for practical use, the [photopolymerization/110°C-thermal decomposition] cycle is a useful way to improve the *PF* of the photopolymerized C_60_ film.

## 4. Other low-dimensional nanocarbons

### 4.1. C_60_ peapods

**Figure 19.**
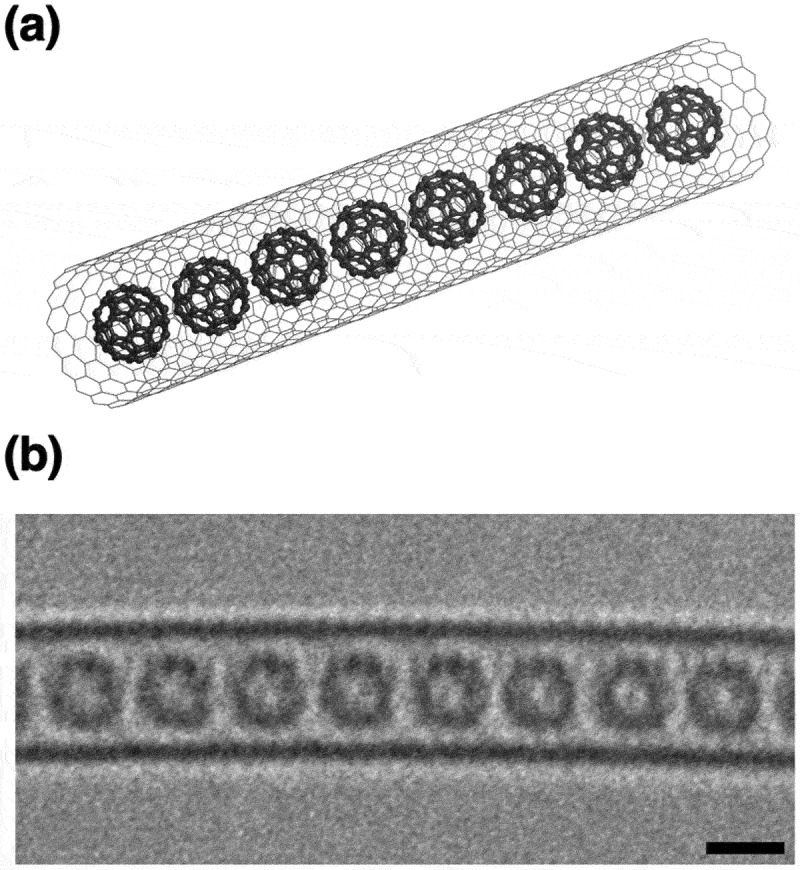
Structure of fullerene peapods: (a) Molecular model of C_60_ molecules encapsulated in (18,0) single-walled CNT, (b) Aberration-corrected TEM image of C_60_ fullerene peapods. Here, the black scale bar is 1 nm.

C_60_ peapods can be regarded as composite materials in which C_60_ molecules are encapsulated within the internal cavities of CNTs (Figure 19(a)), which was first reported in TEM images (Figure 19(b)) of C_60_ molecules aligned in a single-walled CNT (SWCNT) that was accidentally observed by Smith et al. [119]. However, the C_60_ peapods now can be synthesized conveniently with a well reproducibility by using sublimation of C_60_ (dry process) or ‘nano-extraction’ (wet process) technique [120] in the presence of CNTs with open-ended tips. The arrangement of the encapsulated C_60_ molecules can be controlled utilizing the diameter of CNTs used as a container. In particular, when using SWCNTs with a diameter of 1.4 nm, C_60_ molecules with a diameter of 0.7 nm can efficiently achieve van der Waals contact within the internal space of SWCNTs. This results in formation of an ideal 1D C_60_ array (Figure 19(b)).

The distinctive structure of C_60_ peapods has attracted considerable attention from the perspectives of physics and engineering. Especially, their electronic structures, optical, electrical, and magnetic properties have been extensively investigated so far [121]. The impracticality of extracting the 1D C_60_ molecules from the peapods has been focused to examine the properties of C_60_ peapods treated as a C_60_/CNT composite nanocarbon.

Notably, the interactions between CNTs and C_60_ are utilized to control the properties of the CNTs. For instance, the strong electron affinity of C_60_ [105] causes electron transfer from SWCNTs to C_60_ molecules, resulting in a partial positive charge distribution along the axis of SWCNTs. This makes SWCNTs featured as a p-type material. For example, Roth’s group demonstrated a single-electron transistor using C_60_ peapods [122]. In addition, this property has also been reported to enhance a field emission efficiency [123]. Furthermore, the dependence of C_60_ filling ratio on the thermal conductivity of C_60_ peapods has been investigated by equilibrium molecular dynamics (MD) simulations and obtained that the maximum thermal conductivity of C_60_ peapod at filling ratio of 50% is greater by ca. 30 times than that of pristine CNT [124].

In a similar manner to C_60_, C_70_ [125], higher-order fullerene molecules [126], nitrogen-doped fullerenes (N@C59) [127], and metallofullerenes [128] were reported to form 1D arrays as well. Recently, 1D DySc_2_N@C_80_ array in CNTs exhibited a single-molecule magnet [129].

Attempts to produce 1D C_60_ polymers *via* polymerization between the adjacent encapsulated C_60_ molecules in the peapods have been extensively made since the material’s discovery [119]. Fusion induced by EB irradiation was confirmed in the first report [119], and it was shown that fusion reactions between C_60_ molecules progress upon 100 kV EB irradiation, eventually converting the internal 1D C_60_ array in SWCNT to form double-walled carbon nanotubes (DWCNTs). DWCNTs can also be formed from C_60_ peapods under heat treatment at 1200°C [130]. This method is useful to synthesize DWCNTs with precisely controlled diameters at a bulk scale. Another way to polymerize C_60_ in peapods is chemical doping, which emerges a metallic property of the peapods [131].

**Figure 20.**
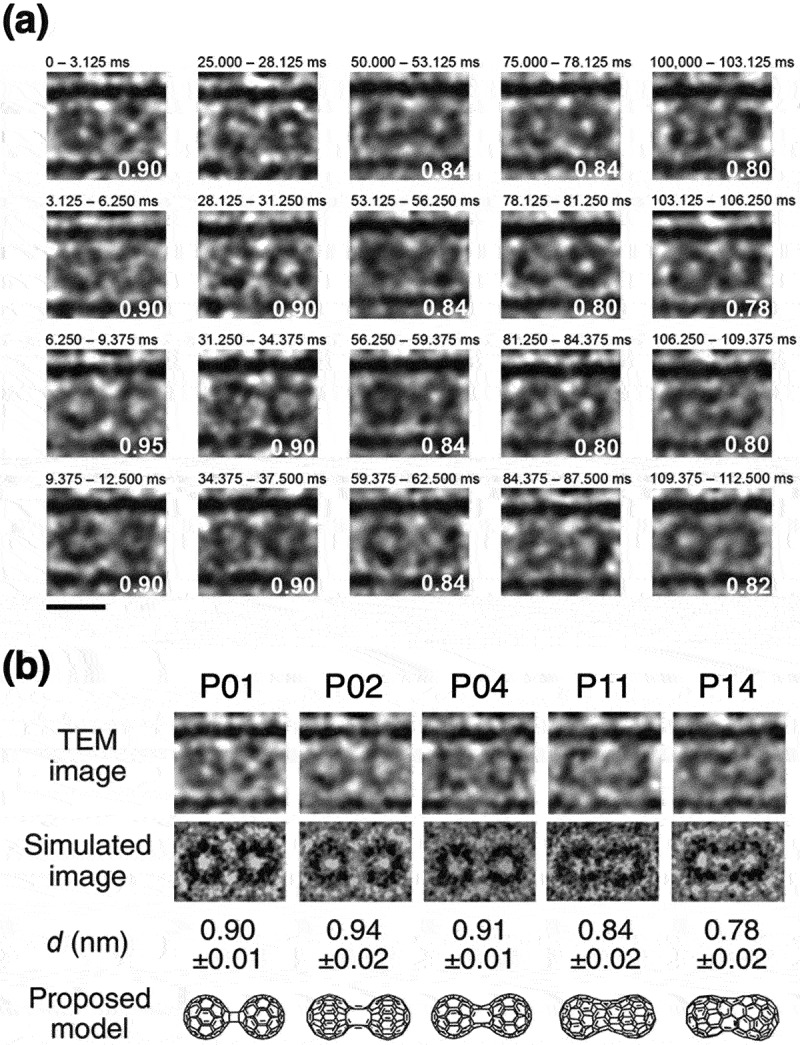
Millisecond-scale TEM video imaging of multistep conversion of C_60_ peapods under electron-beam irradiation [133]: (a) Cinematographic frames (3.125 ms/frame) of a C_60_ dimer undergoing generalized Stone-Wales transformation. The time begins with the start of recording, and the numbers on the right bottom are the intermolecular distances in nm. The scale bar is 1 nm. (b) Experimental, simulated images and proposed models of coalesced dimers for observed intermediates in TEM.

Reprinted with permission from Shimizu et al. [133]. Copyright 2022 American Chemical Society.

When C_60_ peapods are considered as a starting material for the synthesis of 1D C_60_ polymers, CNTs can be used as a test tube in order to track the polymerization reaction of C_60_ using HRTEM [132]. In particular, the development of spherical aberration correctors and high-speed imaging detectors has improved the resolution and frame rates of TEM significantly. This leads to investigate the structure and kinetics of reactions between C_60_ molecules precisely at atomic scale. Nakamura and Harano et al. recorded TEM video with a millisecond time-resolution to capture EB-induced fusion reaction from two C_60_ molecules to one coalesced C_120_ and identified several metastable C_120_ intermediate species based on their shapes and sizes [133]. These reactions occur stochastically, and the lifetime of individual intermediate species varies correspondingly (Figure 20(a)). From the statistical analysis of numerous recorded images of cascade reactions, the average lifetime of various intermediate species was determined. High-energy short-lived intermediate species such as P02 were successfully observed as shown in Figure 20(b). Moreover, by examining both the frequency of individual reaction events in various temperature ranges and the temperature dependence of the reaction rates, it is possible to estimate the *E*_a_ from the excited states [134]. This revealed that the fusion reaction causes *via* the excited states of C_60_ and C_120_ intermediate species that depend on EB kinetic energy [135].

### 4.2. Wavy-structured graphenes

**Figure 21.**
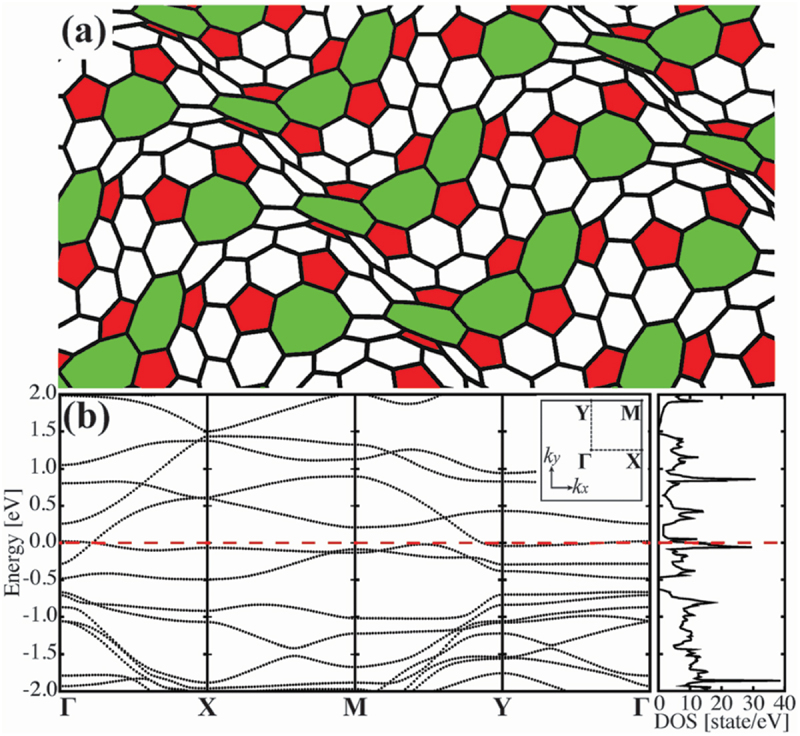
(a) Geometrical structure and (b) Band structure along Γ–X–M–Y–Γ directions and DOS of the wavy-structured graphene with Mickey-Mouse-shaped defects that are periodically aligned parallel to the *y*-direction. The inset in (b) shows the Brillouin zone of the wavy-structured graphene, where fractional coordinates at Γ, X, M, and Y points are given by (*k*_*x*_, *k*_*y*_) = (0.00, 0.00), (0.50, 0.00), (0.50, 0.50), and (0.00, 0.50), respectively. A tilted Dirac cone is located between Γ and X points at the Fermi level. Horizontal dashed red lines in the band structure and DOS indicate the Fermi level [136].

Reprinted with permission from Noda et al. [136]. Copyright 2014 the Royal Society of Chemistry.

Noda et al. theoretically examined wavy-structured graphenes as new metallic carbon allotropes using first-principles calculations based on DFT [136]. Although an ideal graphene is exactly a 2D flat layer atomically, the wavy-structured graphene has convex and concave curved structures induced by the topological defects consisting of 5- and 7-/8-membered rings, respectively. Figure 21(a) shows schematic illustration of the wavy-structured graphene with the defects, which consists of sixty C atoms per unit cell. Such the wavy-structured graphene can be formed from graphene by introducing topological defects *via* the GSW transformation [137,138]. Thus, we examined the effects of the topological defect consisting of pentagon-octagon-pentagon membered rings (named Mickey-Mouse-shaped defect) on the wavy-structured graphene.

Unlike the 2D flat graphene which exhibits strongly dispersive bands intersecting at the Dirac point near *E*_F_, the band structure of the wavy-structured graphene with the Mickey-Mouse-shaped defects shows simultaneous occurrence of both flat and dispersive bands, which are parts of an anisotropic Dirac cone tilted from the isotropic one of the pristine graphene (see Figure 21(b)). Owing to the presence of the flat bands, the wavy-structured graphene has an extremely high DOS near *E*_F_, which is quite similar to that of high-performance metal catalysts such as platinum and palladium [139–141]. As far as we examined, similar characteristics of the flat band and high DOS near *E*_F_ have been found for the other wavy-structured graphene models (see Supplementary Information of Ref. [136]).

### 4.3. Penta-nanotubes

It is well known that CNTs can be obtained from graphene, namely (*n*, *m*) nanotube can be defined using a chiral vector, *C*_*nm*_ = *n**a***_**1**_ + *m**a***_**2**_. Here, ***a***_**1**_ and ***a***_**2**_ are the unit vectors, and *n* and *m* are the integers to indicate the number of unit vectors in graphene. Due to the gapless semi-metallic feature of graphene, the electronic properties of CNTs exhibit a high chirality-dependence. Namely, a CNT is metallic only when its *C*_*nm*_ satisfies (*n*–*m*) = 3*l*, where *l* is an integer. The difficulty in fabricating and separating CNTs with certain conductance (metallic or semiconducting) greatly hinders its application in nanoelectronics.

**Figure 22.**
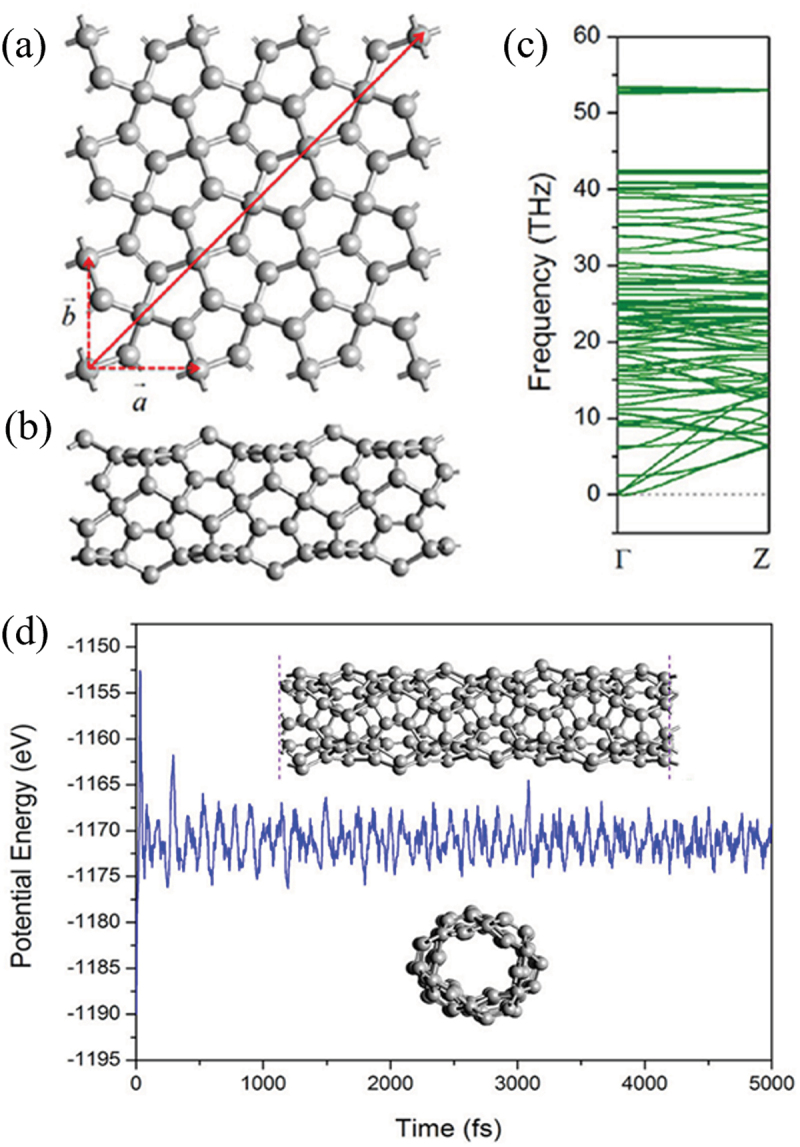
(a) Illustration of chiral vectors of penta-tube. Dashed lines with arrows denote the lattice basis vector. (b) Side view of the optimized structure of (3, 3) penta-tube, (c) The corresponding phonon spectrum，and (d) evolution of potential energy of (3, 3) penta-tube during AIMD simulations at 1000 K, where the insets show snapshots of atomic configuration at the end of the simulation [142].

Going beyond the conventional graphene, penta-graphene exclusively consisting of only 5-membered rings was proposed by Wang et al. [142], which exhibits many interesting properties due to its unique geometric structure (negative Poisson’s ratio, intrinsic piezoelectricity, giant out-of-plane, and second harmonic generation susceptibility) [143,144]. In similar, penta-nanotubes can be constructed by rolling up the penta-graphene sheet along the (*n*, *m*) chiral vectors with *n=m* ranging from 2 to 8, as shown in Figure 22(a) [142]. The optimized geometry of a (3, 3) penta-tube is presented in Figure 22(b). Figure 22(c,d) shows the dynamic and thermal stabilities of this penta-nanotube obtained using phonon spectra calculations and *Ab initio* MD (AIMD) simulations, respectively. All the (*n*, *n*) penta-nanotubes are dynamically robust as well as thermally stable up to 1000 K.

**Figure 23.**
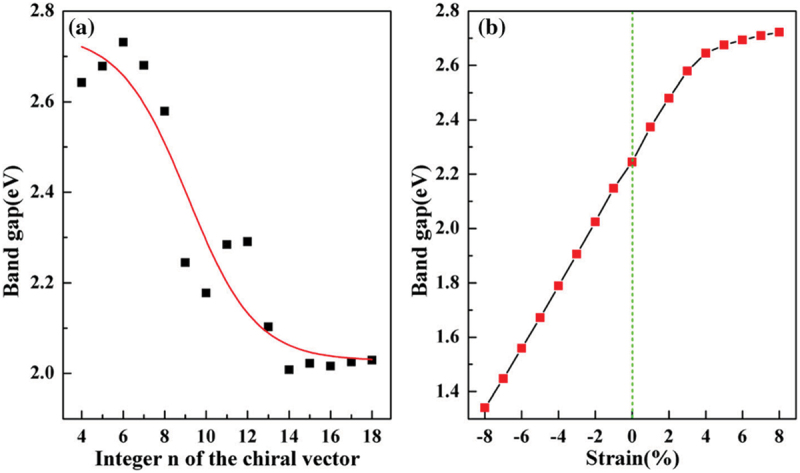
(a) Band gap changing with diameter for (n, n) penta-nanotubes, (b) Strain modulated band gap of (9,9) penta-nanotube [145].

Reprinted with permission from Wang et al. [145]. Copyright 2020 Springer.

More interestingly, the penta-nanotubes inherit the semiconducting feature of penta-graphene. As shown in Figure 23, they are all semiconducting with medium size energy band gaps from 2.008 to 2.731 eV [145]. When the diameter of the inner layer increases from 5.69 Å in (4, 4) to 12.10 Å in (8, 8), the band gap decreases rapidly with increasing the diameter. When the inner diameter is larger than 20.22 Å in (13, 13), the bad gap becomes insensitive to the inner diameter due to the small curvature effects for large penta-nanotubes. This is very different from the conventional CNTs with a chirality-dependent conductance. Namely, they can be metallic or semiconducting that depends on their chirality. This unique feature of penta-nanotube demonstrates the advantages for application to nano electronic devices. In addition, the band gap can be effectively tuned by strain (Figure 23(b) for (9,9) nanotube), where the bad gap reduces linearly under pressive strain, while increasing nonlinearly with stretch.

Mechanically, the elastic modulus of penta-nanotubes was reported to be up to 1000 GPa [146–149], which is comparable to that of conventional CNTs [150]. In addition, several other exceptional features such as the auxeticity at ambient conditions, the ultimate tensile stress of 85‒110 GPa, and the critical strain up to 25% have confirmed the mechanical superiority of penta-nanotubes [146,151] over conventional CNTs.

In contrast to the feature of the bandgap, the fracture patterns of penta-nanotubes strongly depend on the chirality of the nanotubes. For example, Sousa et al. [148] showed that the armchair-type penta-nanotubes are suddenly fractured at lower tensile strain values due to the differences in bonding alignments when compared to zigzag-type penta-nanotubes. Interestingly, penta-nanotubes can have direct *α*-phase and inverted *β*-phase, which depend on the inner dimers being parallel or perpendicular to the main axis of the tube [152]. In addition, strain engineering can be used to improve the structural performance of penta-nanotubes and to enhance their electronic and mechanical tunability under applied conditions. For instance, Wang et al. found that the compressive strain can drive a transition from semiconductor to metal in *β*-phase (4, 4), (5, 5), and (6, 6) tubes [152]. Sousa et al. [146] showed that (11,11) *β*-phase penta-nanotube experiences a structural transition to (11,11) *α*-phase under tensile stretching at RT. In addition, elasticity – plasticity transition can also be induced by tensile strain engineering [149].

Using large-scale MD simulations, Chen et al. [149] found that penta-tube has comparable mechanical properties with that of the conventional CNTs. However, unlike the brittle CNTs, penta-tube exhibits a large extensibility (with failure strain exceeding 60%) and behaves plastically during tensile deformation. The plasticity is inherently caused by the phase transition from pentagonal to polygonal (including trigonal, tetragonal, hexagonal, heptagonal, and octagonal) carbon rings. The plastic feature of penta-tubes depends intrinsically on the tube-diameter and strain-rate.

Recently, Shah et al. [153] reported that porous carbon penta-nanotubes can be used as a sensor for halogen gas (F_2_, Cl_2_, Br_2_, I_2_), where the host carbon atoms were selectively removed to create the nanopores on the tube surface, and the *I – V* curves suggested the performance of gas detection. Furthermore, interaction energy graphs showed the efficient separation of various halogen molecules by functionalizing the pores with F_2_, Cl_2_ and H atoms.

Besides the penta-nanotubes formed from penta-graphene, the other penta-tubes have also been studied. For example, based on the experimental synthesis of penta-PdSe_2_ sheet, Kuklin et al. [154] found that the electronic properties of (n, 0) penta-PdSe_2_ tubes, like that of the penta-PdSe_2_ monolayer, are semiconducting with similar band gaps, whereas (n, n) tubes exhibit indirect – direct band gap transitions following the increase in the tube diameter. Motivated by the recent synthesis of penta-NiN_2_ sheet, Ati et al. [155] explored the H_2_ adsorption on (n, 0) penta-NiN_2_ tubes, and found that when the sheet is curved into nanotubes, the Ni-N distance is enlarged due to the stress, which weakens the orbital hybridizations between Ni and N, and reduces the charge transfer from Ni to N. The Ni ion with a less charge on the tube shows a weak polarizing ability for H_2_ molecule.

The studies on penta-nanotubes have significantly expanded the family of 1D carbon nanomaterials with new features and provided more options for device applications.

## 5. Outlook

**Figure 24.**
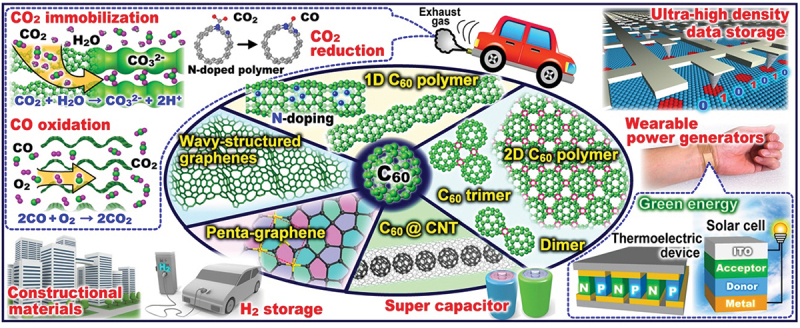
Schematic illustration of C_60_-based low-dimensional nanocarbons in this review: structures and potential applications based on their fundamental properties.

This review has focused on the structures, fundamental properties, and potential applications of low-dimensional C_60_ polymers and related nanocarbons formed *via* photo- and electron-induced polymerization and those of other low-dimensional nanocarbons such as C_60_ peapods, wavy-structured graphenes, and penta-nanotubes, as shown in the summary of Figure 24.

**Figure 25.**
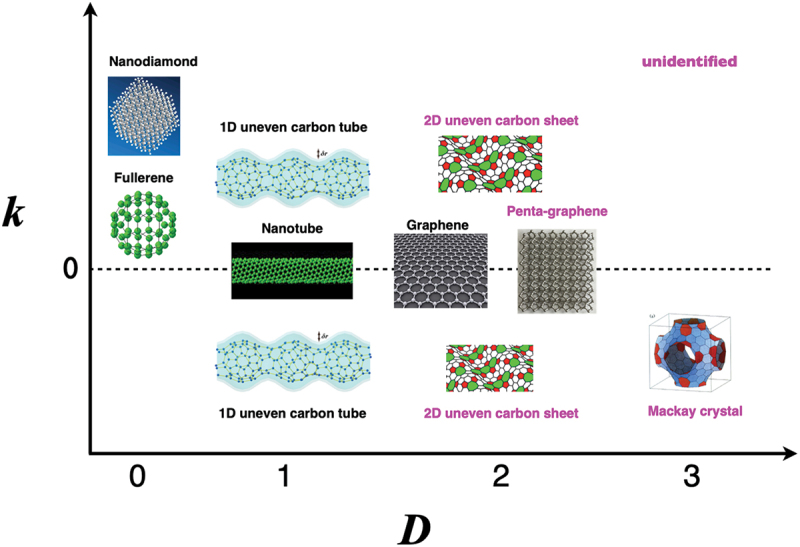
Schematic mapping of low dimensional nanocarbons with respect to Gaussian curvature (*k*) and dimension (*D*), along with 3D Mackay crystal.

As described in Section 2.2, the 1D peanut-shaped C_60_ polymer is the only one material that can examine the properties in curved quantum systems experimentally. This is of great significance to cultivate a new interdisciplinary field between geometry and materials science by pioneering the unexplored doctrine of curved quantum systems (quantum mechanics of submanifolds). More recently, discrete geometry analysis has been applied to several 1D peanut-shaped C_60_ polymers for exploring the correlation between mathematical and physical quantities [156]. Figure 25 shows the mapping of low-dimensional nanocarbons with respect to Gaussian curvature (*k*) and dimension (*D*), along with negatively curved graphitic 3D carbon crystals proposed by Mackay, Terrones, and the other groups [157–159]. Interestingly, Park et al. theoretically predicted to exhibit magnetism due to trivalent carbon radicals in the tetrapod core with a concave surface (Figure 1 of Ref. [160]). As shown in Figure 25, a flexibility of bond direction by sp^2^–sp^3^ combinations further discovers new novel carbon materials different from fullerenes, nanotubes, and graphene.

In our materials world that has hitherto been composed of two axes: physics and chemistry, novel materials have been discovered by ‘knowledge’, ‘experience’, and ‘intuition’ (for example, fullerenes, nanotubes, and graphene) so far. This is so-called ‘Serendipity’. If modern mathematics is incorporated into the materials world as a new axis, novel materials discovered without ‘Serendipity’ (Beyond Serendipity) will no longer be a dream (Figure 3.21 of Ref. [15]).
